# Omega-3-Supplemented Fat Diet Drives Immune Metabolic Response in Visceral Adipose Tissue by Modulating Gut Microbiota in a Mouse Model of Obesity

**DOI:** 10.3390/nu15061404

**Published:** 2023-03-15

**Authors:** Néstor D. Portela, Cristian Galván, Liliana M. Sanmarco, Gastón Bergero, Maria P. Aoki, Roxana C. Cano, Susana A. Pesoa

**Affiliations:** 1 Departamento de Diagnóstico Molecular, LACE Laboratorios, Córdoba X5000JJS, Argentina; 2 Unidad Asociada Área Ciencias Agrarias, Ingeniería, Ciencias Biológicas y de la Salud, Consejo Nacional de Investigaciones Científicas y Tecnológicas (CONICET), Facultad de Ciencias Químicas, Universidad Católica de Córdoba, Córdoba X5016DHK, Argentina; 3 Centro de Investigaciones en Bioquímica Clínica e Inmunología (CIBICI), Consejo Nacional de Investigaciones Científicas y Tecnológicas (CONICET), Córdoba X5000HUA, Argentina; 4 Departamento de Bioquímica Clínica, Facultad de Ciencias Químicas, Universidad Nacional de Córdoba, Córdoba X5000HUA, Argentina

**Keywords:** obesity, immune-metabolism, adipose tissue macrophages, gut microbiota, Omega-3, high fat diet, visceral adipose tissue

## Abstract

Obesity is a chronic, relapsing, and multifactorial disease characterized by excessive accumulation of adipose tissue (AT), and is associated with inflammation mainly in white adipose tissue (WAT) and an increase in pro-inflammatory M1 macrophages and other immune cells. This milieu favors the secretion of cytokines and adipokines, contributing to AT dysfunction (ATD) and metabolic dysregulation. Numerous articles link specific changes in the gut microbiota (GM) to the development of obesity and its associated disorders, highlighting the role of diet, particularly fatty acid composition, in modulating the taxonomic profile. The aim of this study was to analyze the effect of a medium-fat-content diet (11%) supplemented with omega-3 fatty acids (D2) on the development of obesity, and on the composition of the GM compared with a control diet with a low fat content (4%) (D1) over a 6-month period. The effect of omega-3 supplementation on metabolic parameters and the modulation of the immunological microenvironment in visceral adipose tissue (VAT) was also evaluated. Six-weeks-old mice were adapted for two weeks and then divided into two groups of eight mice each: a control group D1 and the experimental group D2. Their body weight was recorded at 0, 4, 12, and 24 weeks post-differential feeding and stool samples were simultaneously collected to determine the GM composition. Four mice per group were sacrificed on week 24 and their VAT was taken to determine the immune cells phenotypes (M1 or M2 macrophages) and inflammatory biomarkers. Blood samples were used to determine the glucose, total LDL and HDL cholesterol LDL, HDL and total cholesterol, triglycerides, liver enzymes, leptin, and adiponectin. Body weight measurement showed significant differences at 4 (D1 = 32.0 ± 2.0 g vs. D2 = 36.2 ± 4.5 g, *p*-value = 0.0339), 12 (D1 = 35.7 ± 4.1 g vs. D2 = 45.3 ± 4.9 g, *p*-value = 0.0009), and 24 weeks (D1 = 37.5 ± 4.7 g vs. D2 = 47.9 ± 4.7, *p*-value = 0.0009). The effects of diet on the GM composition changed over time: in the first 12 weeks, α and β diversity differed considerably according to diet and weight increase. In contrast, at 24 weeks, the composition, although still different between groups D1 and D2, showed changes compared with previous samples, suggesting the beneficial effects of omega-3 fatty acids in D2. With regard to metabolic analysis, the results did not reveal relevant changes in biomarkers in accordance with AT studies showing an anti-inflammatory environment and conserved structure and function, which is in contrast to reported findings for pathogenic obesity. In conclusion, the results suggest that the constant and sustained administration of omega-3 fatty acids induced specific changes in GM composition, mainly with increases in *Lactobacillus* and *Ligilactobacillus* species, which, in turn, modulated the immune metabolic response of AT in this mouse model of obesity.

## 1. Introduction

Obesity is a major public health problem contributing to increased morbidity and mortality worldwide [1]. It is very well known that obesity is a chronic, relapsing, and multifactorial disease representing a risk factor for other non-communicable diseases (NCDs), such as type 2 diabetes mellitus, cardiovascular disease, and cancer, among others [2]. This condition is the consequence of a sustained positive energy balance, leading to excessive accumulation of adipose tissue (AT) [3]. Under physiological conditions, AT plays critical roles in whole-body homeostasis, including the storage and release of energy, thermoregulation, and secretion of adipokines that regulate the energy balance, metabolism, and immune responses [4]. In contrast, obesity is associated with AT inflammation, mainly in white adipose tissue (WAT), showing an increase in pro-inflammatory M1 macrophages and other immune cells, due to tissue remodeling in response to adipocyte apoptosis [5]. This pro-inflammatory milieu favors the secretion of pro-inflammatory cytokines and adipokines, contributing to AT dysfunction (ATD) and metabolic dysregulation [6].

In recent decades, numerous articles have been published linking specific changes in the GM to the development of obesity and its associated disorders [7,8]. The GM is involved in many functions, including nutrient absorption, protection of intestinal mucosal integrity, regulation of immune responses, and being a central regulator of host metabolism [9]. Both structural components, lipopolysaccharides (LPSs) and peptidoglycans, and specific microbial-derived metabolites, such as short-chain fatty acids (SCFA), may act as the central factors in the pathogenesis of obesity and other NCDs [10,11,12]. Remarkably, the GM participates in the development of AT in both normal and pathological conditions. The obesity-associated gut microbiome has an increased capacity to harvest energy from the diet, contributing to increased total body fat, especially VAT [13]. Bäckhed et al. showed that germ-free (GF) mice are protected from diet-induced obesity (DIO) compared with conventional mice, which contained 42% more total body fat [14]. Additionally, it has been shown that certain changes in the abundance and diversity of microbiota are associated with AT inflammation, one of the most significant features of ATD [10]. Different reports have shown that the level of circulating LPS is the key factor linking the GM with the inflammation of AT, resulting in an increase in the number of cells positive for the inflammation marker F4/80 in AT [10,15,16]. Moreover, ATD-associated obesity is characterized by the presence of cellular infiltrates rich in pro-inflammatory M1 macrophages and deficient in anti-inflammatory M2 macrophages [17]. These reports denote the importance of the GM in the regulation of adiposity, the inflammatory state, and the correct functionality in relation to the metabolic state.

It is well established that both the GM structure and function are dynamic and strongly affected by diet nutrients, such as the content and composition of lipids. As a consequence, dietary lipids influence host physiology through interactions with the GM [18], although the mechanisms involved are not well defined. However, it has been shown that high-fat diets (HFDs) decrease the GM diversity and epithelial barrier-protecting bacteria while increasing the abundance of deleterious and pro-inflammatory species [19,20], inducing low-grade chronic inflammation, a phenomenon known as metabolic endotoxemia, which is associated with the development of obesity and numerous NCDs [10,21].

In this context, most of the studies in murine models used diets with a fat content of 60% or higher; these levels accelerated the development of obesity, generating exacerbated metabolic and physiological responses. However, Speakman et al. stated that HFDs with a 60% fat content represent a large distortion of a normal rodent chow, and the results obtained in these studies cannot be potentially extrapolated to humans [22]. Additionally, as mentioned above, different types of dietary fatty acids can differentially influence the GM composition, obesity development, and metabolic manifestations, producing either beneficial or harmful responses [23,24]. Overall, a diet rich in saturated fatty acids (SFAs) promotes low-grade inflammation through direct action on TRL4 [25], reduces the diversity of the GM, increasing the relative abundance of *Firmicutes*, decreasing *Bacteroidota* [26,27], and increasing the proportion of *Proteobacteria* [28]. On the other hand, polyunsaturated fatty acids (PUFAs), a subgroup of essential fatty acids, have differential effects on health and the GM; a diet rich in n-6 PUFA promotes dysbiosis associated with weight gain and the infiltration of macrophages and neutrophils into the ileal submucosae. In contrast, fish oil-supplemented diets rich in eicosapentaenoic acid (EPA) and docosahexaenoic acid (DHA) (n-3 PUFA) restore the GM composition, recruit regulatory T cells (Treg), and decrease the infiltration of macrophages and neutrophils [29]. In addition, n-3 PUFAs have been shown to reduce the production of reactive oxygen species (ROS) and to inhibit the production of pro-inflammatory cytokines, reducing metabolic endotoxemia [30]. However, information on models mimicking, in rodent studies, aspects of the diet that are similar to those found in humans is lacking. As mentioned above, the high fat content in HFD models is not extrapolated to humans. This prompted us to analyze the effect of a medium-fat-content diet (11%) supplemented with omega-3 fatty acids on the development of obesity and its impact on the composition of the GM compared with a control diet with low fat content (4%) over a 6-month period. The proportions of SFAs, monounsaturated fatty acids (MUFAs), and PUFAs were comparable in both diets. Furthermore, we evaluated the contribution of Omega-3 supplementation to metabolic parameters and the modulation of the immunological microenvironment in VAT.

## 2. Materials and Methods

### 2.1. Animals and Experimental Design

Male C57BL/6J (B6) mice (6-weeks-old) were purchased from the Facultad de Ciencias Veterinarias of the Universidad Nacional de La Plata, Buenos Aires, Argentina. All animals were housed in isolation rooms at the Animal Facilities of the Facultad de Ciencias Químicas of the Universidad Católica de Córdoba. This research has the authorization of the Institutional Committee for the Care and Use of Laboratory Animals—CICUAL-FCQ- Universidad Nacional de Córdoba, Córdoba, Argentina. Resolution N° 939, EXP-UNC: 0023836/2018. The environmental conditions in the facility were set according to CICUAL Guidelines. All animal procedures were performed in accordance with the guidelines of Directive 2010/63/EU of the European Parliament on the protection of animals used for scientific purposes.

The mice were randomly divided into two groups of eight mice each and adapted for two weeks to the experimental conditions. Then, the mice were fed a low-fat diet, “Control Diet” (D1, 4% fat), or an “obesity-induced diet” with medium fat content (D2, 11% fat) for a period of 24 weeks. Both groups were maintained under a standard light cycle (12 h light/dark), with free access to water and food, and temperature conditions (21 ± 2 °C). The food was replaced every two days to keep it fresh.

### 2.2. Evaluation of Development of Obesity

The body weight of the mice was evaluated at 0, 4, 12, and 24 weeks. DIO was defined by the cut-off point of the mean body weights of the mice at week 0 plus 3 standard deviations in both groups (D1 and D2).

### 2.3. Food Composition

Control Diet composition (D1): 4% fat, 26% protein, 52% carbohydrate, 8% crude fiber, and 10% total minerals (GEPSA Pet Foods, Pilar, Argentina). Obesity-Inducing Diet composition (D2): 11% fat, 27% protein, 48% carbohydrate, 6% crude fiber, and 8% total minerals. Ingredients: Corn, wheat, rice, soybean meal, chicken by-product meal, beef fat and/or chicken oil preserved with mixed tocopherols, beef and egg meal, corn and/or wheat gluten meal, fish flour (natural source of DHA), and animal digest based on chicken and/or pork by-products and salt (Purina Nestlé). The lipid composition was as follows: D1, SFAs: 24.61%, MUFAs: 39.37%, PUFAs: 36.02%, omega-3 fatty acids: 20.29%, omega-6 fatty acids: 15.73%, and omega-3/omega-6 ratio: 1.29; and D2, SFAs: 25.81%, MUFAs: 35.38%, PUFAs: 38.81%, omega-3 fatty acids: 30.14%, omega-6 fatty acids: 8.67%, and omega-3/omega-6 ratio: 3.48.

### 2.4. Food Intake Assay

To assess the acceptance of both the D1 diet and D2 diet, we carried out a consumption assay to measure food intake by mice. Briefly, we used individual mouse cages (*n* = 10/each diet) with one mouse each in the animal laboratory facility under the conditions cited above under Animals and Experimental Design. The initial amount of feed was weighed (5 g/cage to record daily feed intake). Consumption was measured by the differential weighing of the food at 0 and 24 h after feeding using an Ohaus digital scale model CS200, precision ± 0.1 g. Two independent experiments were performed to obtain an acceptable trend prior to the statistical analysis of the data (*p*-value < 0.05 was considered statistically significant) [31].

### 2.5. Analysis of Gut Microbiota Composition

The GM composition was studied in stool samples, collected individually in metabolic cages over a period of 6 h at weeks 0, 4, 12, and 24. The feces were frozen immediately after collection and stored at −40 °C until analysis.

### 2.6. DNA Extraction

Stool samples were handled under a laminar flow hood using a sterile technique. Microbial DNA was isolated from 220 mg of stool using the QIAmp DNA Stool Mini Kit (Qiagen, Germantown, MD, USA) following the manufacturer’s standard protocol. The DNA concentrations were measured using fluorometric quantitation with a Qubit 2 and the Qubit dsDNA high-sensitivity kit (Thermo Fisher Scientific, Carlsbad, CA, USA). The DNA was stored at −40 °C.

### 2.7. 16S rRNA Gene Amplicon Library Preparation Sequencing and Taxonomic Identification of Bacteria

Sequencing was performed using an Ion 16S Metagenomics Kit (Thermo Fisher Scientific, Carlsbad, CA, USA) on the Ion Torrent Personal Genome Machine (PGM) platform (Thermo Fisher Scientific, Carlsbad, CA, USA). The libraries were generated from 20 ng of fecal DNA with the Ion 16S Metagenomics Kit using a combination of two pools of primers targeting the V2, V4, and V8 hypervariable regions of the 16S rRNA gene in pool 1, and the V3, V6-7, and V9 regions in pool 2. The primers were partially digested and bar-codes were ligated to the amplicons, purified using the Agencourt AMPure XP beads (Beckman Coulter; Pasadena, CA, USA) according to the manufacturer’s protocol, and stored at −20 °C. The concentration of each 16S library was determined by qPCR using the Ion Universal Library Quantitation Kit (Thermo Fisher Scientific Carlsbad, CA, USA). The library was diluted to ~10 pM before template preparation. Template preparation of the barcoded libraries was performed using the Ion PGM Hi-Q View OT2 kit (Thermo Fisher Scientific Carlsbad, CA, USA) and the Ion OneTouch 2 System (Thermo Fisher Scientific Carlsbad, CA, USA). A mock community dataset was generated from mixed bacterial genomic DNA from ATCC strains, including *Escherichia coli ATCC 25922*, *Staphylococcus aureus ATCC 25923*, *Pseudomonas aeruginosa ATCC 27853*, *Enterococcus faecalis ATCC 29212*, and *Streptococcus group B*; the latter was isolated and typified in the LACE Laboratory. A maximum of 12 barcoded 16S samples were sequenced on an Ion 316v2 chip using the Ion PGM Hi-Q view Sequencing Kit (Thermo Fisher Scientific; Carlsbad, CA, USA) according to the manufacturer’s instructions.

The sequence quality control, annotation, and taxonomical assignment were performed using the DADA2 v1.22.0 [32], phyloseq v1.38.0 [33], and microbiome v1.16.0 [34] packages in R software v4.1.2 [35] following the standard pipeline from demultiplexed fastq files. DADA2-formatted Silva Database Version 138.1–Updated 10 March 2021, was used for taxonomical assignment [36]. For the generation of functional profiles based on the composition of the GM, the Tax4Fun2 package v 1.1.5 [37] was used on the R platform for genus-level taxonomy. Linear discriminant analysis Effect Size (LEfSe), performed using an online tool on the galaxy platform (http://huttenhower.sph.harvard.edu/galaxy/) accessed on 28 November 2022 [38], was applied to determine the existence of differential characteristics between the study groups for taxonomy in the GM or for the predicted functional profiles. Sequencing data are accessible in the National Center for Biotechnology Information (NCBI) database under BioProject accession number PRJNA929200 (https://ncbi.nlm.nih.gov/bioproject/?term=PRJNA929200) accessed on 29 January 2023.

### 2.8. Evaluation of Blood Metabolic Profile and Immune Cells Population in VAT

After 24 weeks of differential feeding, 4 mice per group were randomly selected and anesthetized using inhaled FORANE (isoflurane). Blood samples were obtained after 12 h of fasting by cardiac puncture in heparinized tubes, centrifuged at 3000 rpm, and the separated plasma was stored at −20 °C. The plasma triglyceride (TG, mg/dL), total cholesterol (TC, mg/dL), HDL-Cholesterol (HDL-c, mg/dL), glucose (Glu, mg/dL), aspartate aminotransferase activity (AST, U/L), and alanine aminotransferase activity (ALT, U/L) levels were assessed using enzymatic kits (Roche Diagnostic) in a ROCHE Cobas 8000 auto-analyzer; and LDL-Cholesterol (LDL-c) was estimated using the Friedewald formula as: (LDL-c = TC − [HDL-c + TG/5]). The adiponectin and leptin levels were quantified using an Adiponectin Mouse ELISA Kit (Abcam) and Leptin Mouse ELISA Kit (Invitrogen), respectively, following the manufacturer’s instructions. The mice were then sacrificed by cervical dislocation and the VAT was kept for studies of cell populations in stromal vascular fraction (SVF) as described below.

### 2.9. Isolation of the SVF from Adipose Tissue

Mouse epididymal AT was processed by mechanical degradation and digested for 45 min at 37 °C with type 2 collagenase (0.8 mg/mL; Sigma) in Hanks’ Balanced Salt solution (pH = 7.4). After the addition of 3 vol. PBS containing 5% FBS and filtration of the digested tissue through nylon mesh (70 μm), the filtrate was centrifuged at 200× *g*. The SVF was recovered from the resulting supernatant [39].

### 2.10. Flow Cytometry

The SVF of mouse epididymal AT was prepared as described above. Red blood cells were separated by centrifugation at 500× *g* for 5 min, and the remaining cells were suspended in PBS and exposed to FcBLOCK (BD Biosciences) for 20 min. Five hundred thousand SVF cells were washed in ice-cold FACS buffer (PBS-2%FBS) and incubated with fluorochrome-labeled antibodies for 30 min at 4 °C. Different combinations of the following antibodies were used: PeCy5-labeled: anti-CD11b, PE-labeled: anti-F4/80, APCCy7-labeled: anti-CD11c, PeCy7-labeled: anti-CD206, and APC/Alexa 647-labeled: anti-CD36. Cells were permeabilized with BD Cytofix/Cytoperm and Perm/Wash (BD Biosciences) to detect intracellular ROS according to the manufacturer’s instructions. Then, the cells were incubated with FITC/Alexa 488-labeled antibody for ROS. The cells were acquired on FACS Canto II (BD Bioscience). The results were expressed as the percentage of cells per gram of AT.

### 2.11. Statistical Data Analysis

Statistical analysis was carried out and visualized using R v4.1.2 software [35]. The alpha diversity (observed ASVs, and Shannon and Simpson indexes) and beta diversity (PCA and UniFrac, weighted and unweighted) were calculated based on the ASV table representing the relative abundances of bacterial taxa from the microbiome v1.6.0 R package [34]. The association between diets and the overall microbiota composition was tested using the Adonis test through the Adonis function in the vegan v2.4.6 R package [40]. The normality of the variables was assessed using the Shapiro–Wilk test. A *p*-value of < 0.05 was considered significant. Comparative and differential analysis between variables was performed using the two-tailed Student’s *t* test for variables with a normal distribution (result expressed as the mean ± standard deviation (SD)), and the Wilcoxon or Friedman tests for variables without a normal distribution (result expressed as the median, min., and max.), as appropriate. Pairwise comparisons using the paired Wilcoxon signed-rank test were performed if the Friedman test yielded a significant result. The *p*-values were adjusted using the Bonferroni multiple-testing correction method. Correlations between the bacterial taxa’s abundances and metabolic parameters and cell populations in SVF were calculated using the corrplot package v 0.92 [41]. For Lefse analysis, LDA scores of 2 and a *p*-value of <0.05 were considered significant. All data were represented using ggplot2 v3.4.0 [42] and ggpubr v0.5.0 [43].

## 3. Results

### 3.1. Effect of Diets on Body Weight

**Figure 1:**
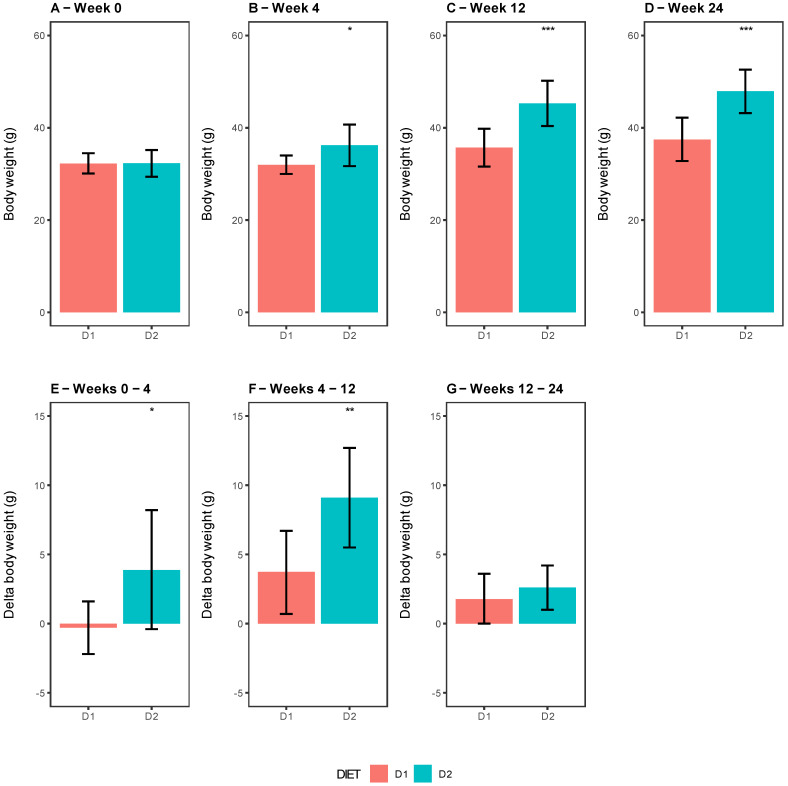
(**A**–**D**) Body weight comparison between groups D1 and D2 by time. Bar plots representing the body weight of each group, Group D1 (pink) and Group D2 (sky blue), for weeks 0 (**A**), 4 (**B**), 12 (**C**), and 24 (**D**). (**E**–**G**) Bar plots representing the delta weights between different weeks for Group D1 (pink) and Group D2 (sky blue) at weeks 0–4 (**E**), weeks 4–12 (**F**), and weeks 12–24 (**G**). Data are shown as the mean ± SD of eight mice per group. Significant differences were established using the two-tailed Student’s *t* test (*: *p* ≤ 0.05, **: *p* ≤ 0.01, ***: *p* ≤ 0.001).

As can be seen in Figure 1, the body weight in mice from Group D2 progressively increased along weeks 4, 12, and 24 compared with those from Group D1, and 25, 87.5, and 100% of mice respectively presented DIO in Group 2 (Figure 1B–D). Remarkably, the weight gain, considered as the delta weight between different weeks, showed a significant difference between groups, being higher in D2-fed mice at weeks 4 and 12 (*p*-value = 0.0331 and 0.0059), not showing a significant change after 24 weeks of feeding (*p*-value = 0.3438), respectively (Figure 1E–G). Is important to mention that no significant differences were observed in food intake between the groups (D1 = 2.7 ± 0.5 g/24 h vs. D2 = 2.9 ± 0.2 g/24 h, *p*-value 0.5747).

### 3.2. Gut Microbiota Analysis

#### 3.2.1. Alpha and Beta Diversity within and between Diet Groups

**Figure 2:**
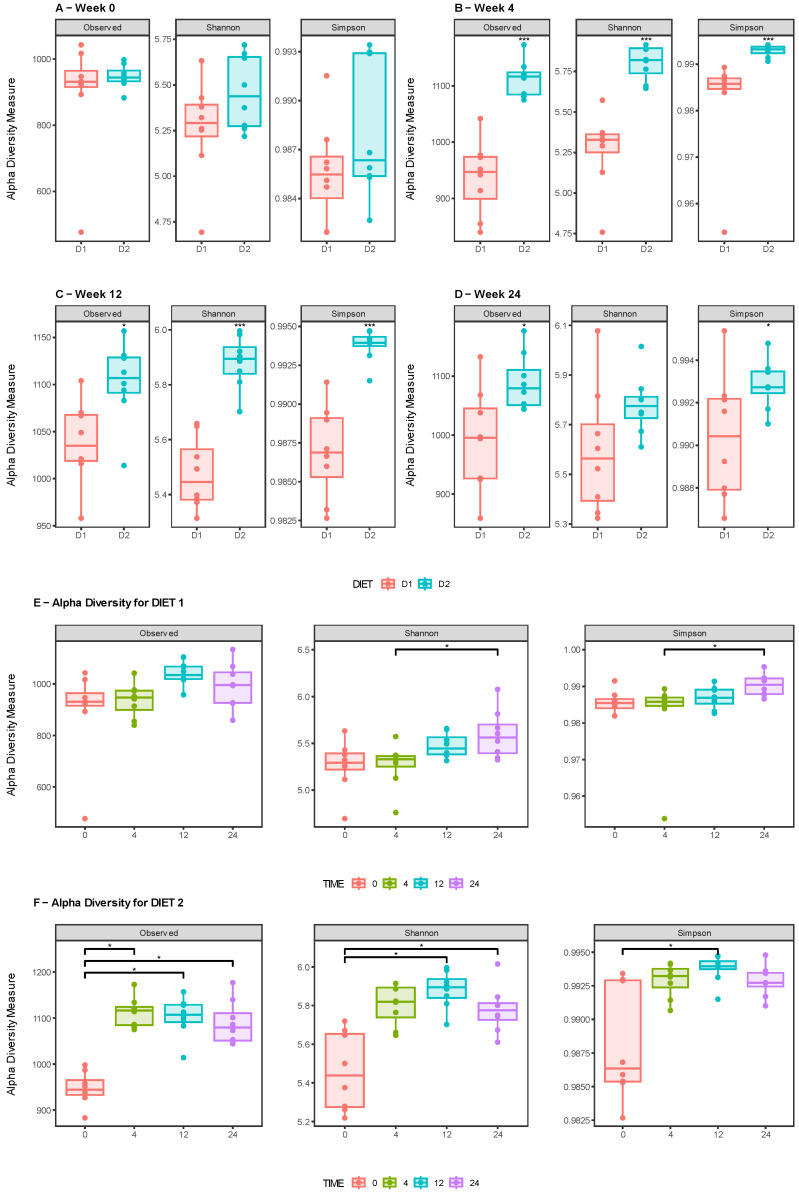
Analysis of alpha diversity between diet groups. Box plots showing observed ASV index, Shannon index, and Simpson index of each group, Group D1 (pink) and Group D2 (sky blue), for weeks 0 (**A**), 4 (**B**), 12 (**C**), and 24 (**D**). (**E**,**F**) Analysis of alpha diversity within Groups D1 and D2 at week 0 (pink), week 4 (green), week 12 (sky blue), and week 24 (purple). The solid black lines indicate the medians, and the lower and upper bounds of the box represent the 25 and 75% quartiles. Outliers are indicated as black circles and represent samples falling outside the 10 and 90% quartiles. Significant differences were established using the Wilcoxon signed-rank test (*: *p* ≤ 0.05, ***: *p* ≤ 0.001).

The comparative analysis of alpha diversity, measured as observed ASVs, Shannon and Simpson Indexes, between Groups D1 and D2 at weeks 0, 4, 12, and 24 is shown in Figure 2. There were no differences at time 0, but mice from Group D2 showed a significant increase compared with those from Group D1 at weeks 4 and 12; likewise, at week 24, observed ASVs and Simpson index had higher values in Group D2 compared with Group D1, in contrast to Shannon Index, which showed a similar trend, although the differences were not statistically significant.

The analysis within Group D1 only showed significant differences for Shannon and Simpson indexes when comparing week 4 and week 24 (Figure 2E). In contrast, in Group D2, significant differences in observed ASVs were found when comparing week 0 with weeks 4, 12, and 24. A similar pattern was seen for Shannon Index at weeks 12 and 24, while Simpson Index was only higher at week 12 in relation to week 0. (Figure 2F). Supplementary Tables S1A–C show statistical comparisons of the alpha diversity indexes.

#### 3.2.2. Beta-Diversity Analysis

**Figure 3:**
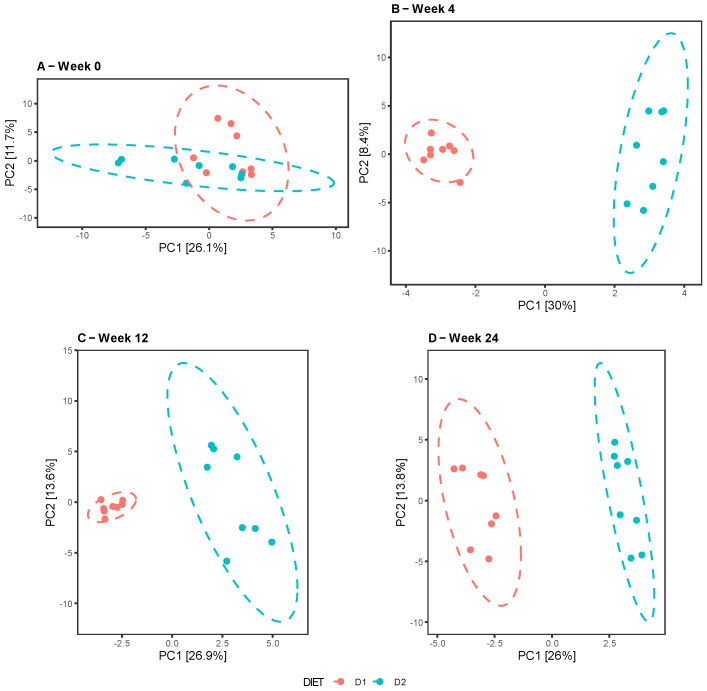
Beta diversity analysis. Comparison of the microbiota profiles of Group D1 (pink) and Group D2 (sky blue) using principal component analysis (PCA). The first two principal components, PC1 and PC2, were plotted for weeks 0 (**A**), 4 (**B**), 12 (**C**), and 24 (**D**).

**Figure 4:**
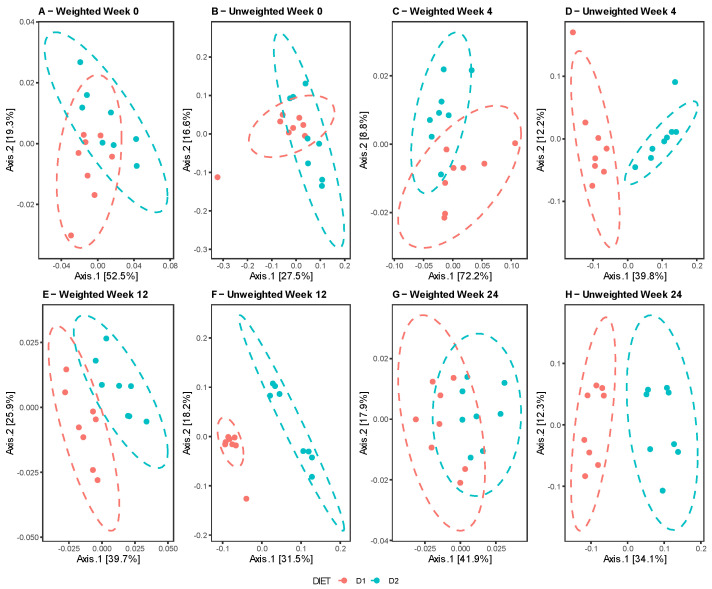
Beta diversity analysis. Comparison of the microbiota profiles of Group D1 (pink) and Group D2 (sky blue) based on the weighted (**A**, **C**, **E** and **G**) and unweighted (**B**, **D**, **F** and **H**) UniFrac distances for weeks 0, 4, 12, and 24, respectively.

The comparative analysis between diets at different times revealed no differences at week 0, in contrast to weeks 4, 12, and 24, when the groups clustered separately, indicating dissimilarities in the composition and taxonomic abundance of their microbiota (Figure 3A–D). Similar results were reported by the Unifrac test in both the weighted and unweighted analyses, with the exception of week 24, showing a certain overlap of the clusters for the weighted Unifrac in contrast to the unweighted Unifrac, denoting dissimilarity between the groups, probably due to the presence of taxa in low abundance. (Figure 4A–H).

#### 3.2.3. Analysis of Relative Abundances at the Phylum and Genus Levels

**Figure 5:**
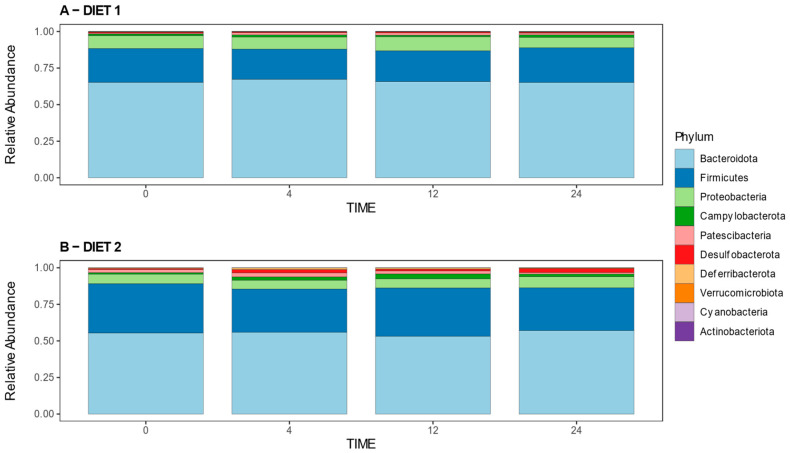
Effect of diet on the gut microbiota composition over time. Bar plot representing the microbiota composition at the phylum level in diets D1 (**A**) and D2 (**B**) at weeks 0, 4, 12, and 24.

Figure 5 illustrates the relative abundances of bacterial phyla per group at different times. Statistical analysis revealed no differences at week 0; a higher abundance of the phyla *Campylobacterota*, *Cyanobacteria*, *Deferribacterota*, *Desulfobacterota,* and *Firmicutes* and lower abundance of *Bacteroidota* and *Proteobacteria* in Group D2 compared with D1 at week 4; different changes were detected at week 12, when D2 presented a higher prevalence of *Deferribacterota*, *Desulfobacterota*, and *Firmicutes* and lower prevalence of *Actinobacterota*, *Bacteroidota*, and *Proteobacteria* compared with Group D1; meanwhile, at week 24, Group D2 showed a higher abundance of *Desulfobacterota* and *Pastescibacteria* and lower abundance of the *Cyanobacteriota* Phylum in comparison with the control group. Supplementary Table S2 shows the statistical comparison of the relative abundances at the phylum level.

**Figure 6:**
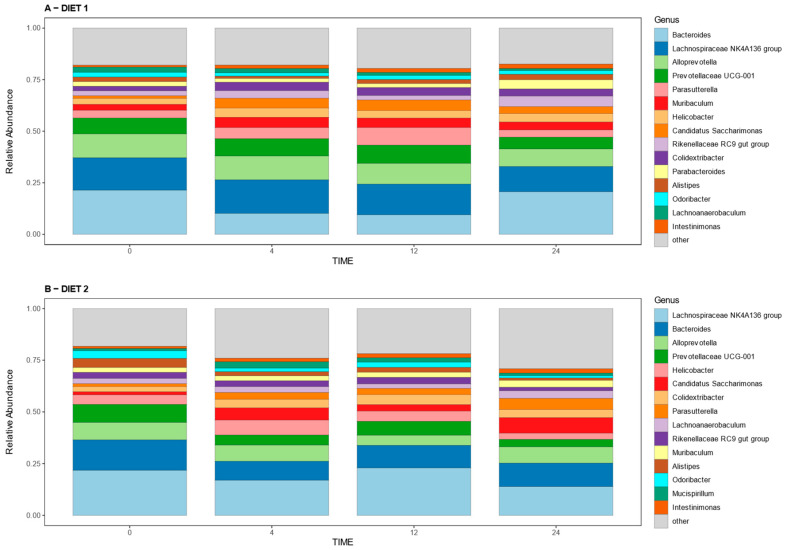
Effect of diet on the gut microbiota composition over time. Bar plot representing the microbiota composition at genus level in diets D1 (**A**) and D2 (**B**) at weeks 0, 4, 12, and 24. Only the 15 main genera found in all samples at all times are shown. This represents more than the 70 percent of the relative abundance of the total genera detected in the samples. The remaining genera are collectively represented as other.

Figure 6 shows the 15 main genera found in all samples at all times, which represent more than 70 percent of the relative abundance of the total genera detected in the samples. The comparative analysis for genera shows higher relative abundances of bacteria from the Eubacterium eligens group, Lachnospiraceae UC5-1-2E3, and Mycoplasma genus for Group D2 at week 0; while the abundances of Escherichia-Shigella and Olsenella were higher in group D1, these genera were not present among the more abundant showed in Figure 6. A higher abundance of Helicobacter and Alistipes, and lower abundance of Prevotellaceae UCG-001 and Muribaculum was seen at week 4 in Group D2 compared with D1; meanwhile, at week 12, a higher prevalence of Lachnospiraceae NK4A136 group and lower prevalence of Parasutterella and Muribaculum was detected in group D2 compared with group D1; these genera were present within the main genera defined above. At week 24, Group D2 had a higher proportion of the genera Lachnoanaerobaculum, Candidatus Saccharimonas, and Ligilactobacillus, and a lower proportion of Alistipes compared with Group D1. Supplementary Table S3 shows the statistical comparison of the relative abundances at the genus level.

### 3.3. LEfSe Differential Analysis

To determine which taxa were enriched in the different groups, linear discriminant analysis (LDA) coupled with effect size measurements (LEfSe) was applied. First, the effect of diet in each sampling week was compared and, later, each group was analyzed over time to determine specific changes not associated with diet. Significant differences at the levels of phylum, class, order, family, and genus were found among the different groups. Only enriched genera will be mentioned below to simplify interpretation.

#### 3.3.1. LEfSe Analysis by Time

**Figure 7:**
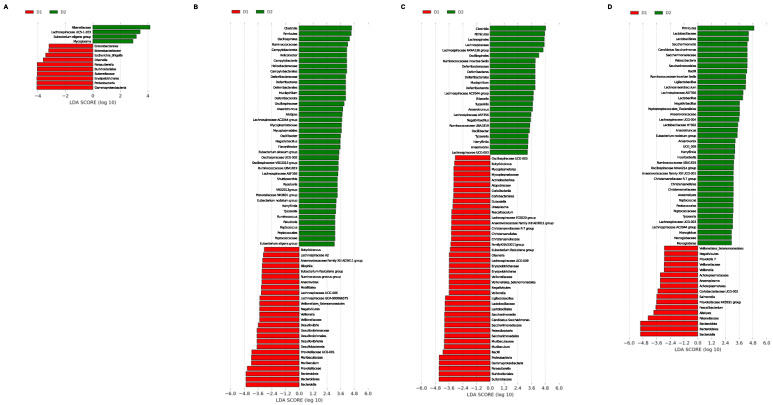
Linear discriminant analysis (LDA) coupled with effect size measurements (LEfSe) by time. The bar plots represent the significantly different taxa between Group D2 (green) and Group D1 (red) at weeks 0 (**A**), 4 (**B**), 12 (**C**), and 24 (**D**). Enriched taxa in Group D2 are represented by a positive LDA score and enriched taxa in Group D1 are represented by a negative LDA score. LDA scores > 2 and *p*-value < 0.05 obtained by the Kruskal–Wallis test were considered significant.

These results are shown in Figure 7. At week 0, group D1 presented a high proportion of *Escherichia/Shigella*, *Orsenella*, and *Parasutterella* in contrast to group D2, presenting higher abundance of the *Eubacterium eligens group*, *Mycoplasma*, and *Lachnospiraceae UC5-1-2E2* genera (Figure 7A). At week 4, the most relevant genera were *Butyricicoccus*, *Lachnospiraceae A2*, *Anaerovoracaceae Family XIII AD3011 group*, *Eubacterium fissicatena group*, *Ruminococcus gnavus group*, *Anaerovorax*, *Mobilitalea*, *Lachnospiraceae UCG-006*, *Lachnospiraceae GCA-900066575*, *Veillonella*, *Desulfovibrio*, *Prevotellaceae UCG-001*, and *Muribaculum* for D1; and *Helicobacter*, *Mucispirillum*, *Anaerotruncus*, *Alistipes*, *Lachnospiraceae AC2044 group*, *Oscillibacter*, *Flavonifractor*, *Eubacterium siraeum group*, *Oscillospiraceae UCG-002*, *Oscillospira*, *Ruminococcaceae UBA1819*, *Lachnospiraceae ASF356*, *Shuttlewoethia*, *Roseburia*, *Prevotellaceae NK3B31 group*, *Oscillospiraceae V9D2013 group*, *Eubacterium nodatum group*, *Harryflintia*, *Tyzzerella*, *Ruminococcus*, *Paladicola*, *Peptococus*, and *Eubacterium eligens group* for Diet D2 (Figure 7B). *Oscillospiraceae UCG-003*, *Butycicicoccus*, *Dobosiella*, *Ureaplasma*, *Faecalibaculum*, *Lachnospiraceae FCS020 group*, *Anaerosporobacter*, *Christensenellaceae R-7 group*, *Anaerovoracaceae Family XIII AD3011 group*, *Eubacterium fissicatena group*, *Orsenella*, *Lachnospiraceae UCG-009*, *Veillonella*, *Ligilactobacillus*, *Candidatus Saccharimonas*, *Muribaculum*, and *Sutterella* were relevant for D1 at week 12, while the genera *Lachnospiraceae NK4A136 group*, *Mucispirillum*, *Lachnospiraceae AC2044 group*, *Rikenella*, *Tuzzerella*, *Anaerotruncus*, *Lachnospiraceae ASF356*, *Ruminococcaceae UBA1819*, *Oscillibacter*, *Harryflintia*, *Tyzzerella*, *Anaerovorax*, and *Lachnospiraceae UCG-003* characterized group D2 (Figure 7C). Finally, LEfSe analysis revealed that the abundances of *Prevotella 7*, *Veillonella*, *Anaeroplasma*, *Coriobacteriaceae UCG-002*, *Salmonella*, *Prevotellaceae NK3B31 group*, *Faecalibacterium*, and *Alistipes* on one hand, and *Candidatus Saccharimonas*, *Ligilactobacillus*, *Lachnospiraceae ASF356*, *Lactobacillus*, *Lachnospiraceae UCG-004*, *Lactobacillaceae HT002*, *Anaerotruncus*, *Eubacterium nodatum group*, *Harryflintia*, *Ruminococcaceae UBA1819*, *Oscillospiraceae NK4A214 group*, *Anaerovoracaceae Family XIII UCG-001*, *Christensenellaceae R-7 group*, *Anaerostipes*, *Peptococcus*, *Tyzzerella*, *Lachnospiraceae UCG-003*, *Lachnospiraceae AC2044 group*, and *Monoglobus* on the other allowed for the best characterization of groups D1 and D2, respectively (Figure 7D).

Overall, these results show that the genera *Harryflintia*, *Lachnospiraceae AC2044 group*, *Lachnospiraceae ASF356*, *Mucispirillum*, *Ruminococcaceae UBA1819*, and *Tyzzerella* could be considered a microbial signature for GM in mice fed with a fatty acid-rich diet, as they presented the same distribution profile in the differential analysis between diets over time. In contrast, the *Eubacterium fissicatena group*, *Anaerovoracaceae Family XIII AD3011 group*, *Butyricicoccus*, *Muribaculum*, and *Veillonella* genera could represent a microbial signature for the chow diet, although just for weeks 4 and 12, when they presented similar distribution patterns. These observations confirm that there was not a single taxon or few taxa allowing the prediction of changes associated with eating patterns involving fatty diets in GM.

#### 3.3.2. LEfSe Analysis by Diet over Time

**Figure 8:**
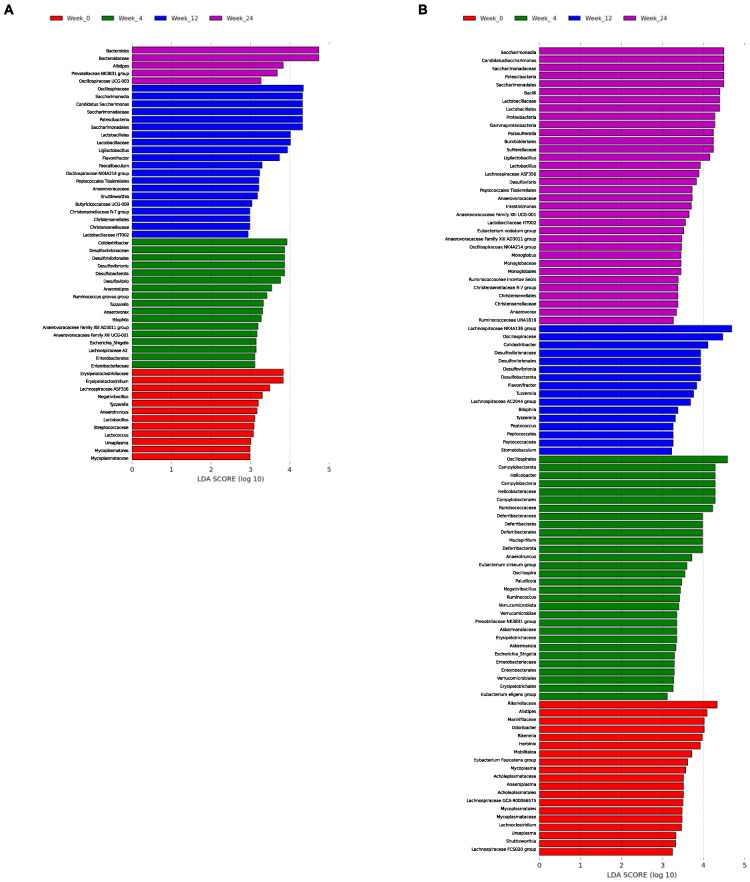
Linear discriminant analysis (LDA) coupled with effect size measurements (LEfSe) by diet. The bar plots represent the significantly different taxa among week 0 (red), week 4 (green), week 12 (blue), and week 24 (purple) in Diet D1 (**A**) and Diet D2 (**B**). LDA scores > 2 and *p*-value < 0.05 obtained by the Kruskal–Wallis test were considered significant.

The results are shown in Figure 8. The analysis over time for D1 revealed that *Lachnospiraceae ASF356*, *Negativibacillus*, *Tyzzerella*, *Anaerotruncus*, *Lactobacillus*, *Lactococcus*, and *Ureaplasma* were the main genera allowing the discrimination of the basal state of the group; at week 4, *Colidextribacter*, *Desulfovibrio*, *Anaerostipes*, *Ruminococcus gnavus group*, *Tuzzerella*, *Anaeroborax*, *Bilophila*, *Anaerovoracaceae Family XIII AD3011 group*, *Anaerovoracaceae Family XIII UCG-001*, *Eschericha-Shigella*, and *Lachnospiraceae A2* described the gut microbial community. *Candidatus Saccharimonas*, *Ligilactobacillus*, *Flavonifractor*, *Faecalibaculum*, *Oscillospiraceae NK4A214 group*, *Shuttlewoethia*, *Butyricicoccaceae UCG-009*, *Lactobacillaceae HT002* and *Christensenellaceae R-7 group* were predominant at week 12. Finally, *Bacteroides*, *Alistipes*, *Prevotellaceae NK3B31 group*, and *Oscillospiraceae UCG-003* allowed the prediction of the phylotype at week 24 (Figure 8A). The study of D2 showed that *Alistipes*, *Odoribacter*, *Rikenella*, *Herbinix*, *Mobilitalea*, *Eubacterium fissicatena group*, *Mycoplasma*, *Anaeroplasma*, *Lachnospiraceae GCA-900066575*, *Ureaplasma*, *Shuttleworthiam*, and *Lachnospiraceae FCS020 group* described the community for week 0. *Helicobacter*, *Mucispirillum*, *Anaerotruncus*, *Eubacterium siraeum group*, *Oscillospira*, *Paludicola*, *Negatibacillus*, *Ruminococcus*, *Prevotellaceae NK3B31 group*, *Akkermansia*, *Escherichia-Shigella*, and *Eubacterium eligens group* characterized week 4. *Lachnospiraceae NK4A136 group*, *Colidestribacter*, *Flavonifractor*, *Tuzzerella*, *Lachnospiraceae AC2044 group*, *Bilophila*, *Tyzzerella*, and *Peptococcus* were predominant at week 12. Finally, *Candidatus Saccharimonas*, *Parasutterella*, *Ligilactobacillus*, *Lactobacillus*, *Lachnospiraceae ASF356*, *Desulfovibrio*, *Intestinimonas*, *Anaerovoracaceae Family XIII UCG-001*, *Lactobacillaceae HT002*, *Eubacterium nodatum group*, *Anaerovoracaceae Family XIII AD3011 group*, *Oscillospiraceae NK4A214 group*, *Monoglobus*, *Ruminococcaceae Incertae Sedis*, *Christensenellaceae R-7 group*, *Anaeroborax*, and *Ruminococcaceae UBA1819* were more abundant at week 24 (Figure 8B).

### 3.4. Prediction of Metabolic Pathways

**Figure 9:**
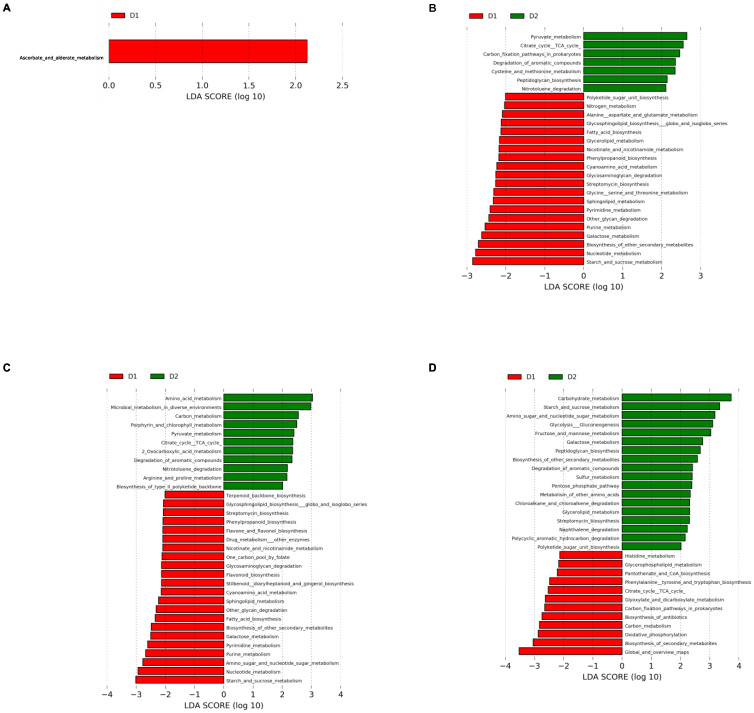
Linear discriminant analysis (LDA) coupled with effect size measurements (LEfSe) for predicted metabolic pathways. The bar plots represent the significantly different metabolic pathways between Group D2 (green) and Group D1 (red) at weeks 0 (**A**), 4 (**B**), 12 (**C**), and 24 (**D**). Enriched metabolic pathways in Group D2 are represented by a positive LDA score and enriched metabolic pathways in Group D1 are represented by a negative LDA score. LDA scores > 2 and *p*-value < 0.05 obtained by the Kruskal–Wallis test were considered significant.

To explore the metabolic pathways associated with different gut microbial communities over time, we carried out functional metagenome prediction using Tax4fun2. This approach allowed the identification of 151 metabolic pathways whose abundances were compared between diet groups at each week tested. LEfSe was applied to determine which metabolic pathways were enriched in the different groups. The results are presented in Figure 9.

The ascorbate and aldarate metabolism pathway from carbohydrates metabolism were more abundant in group D1 than in group D2 at time 0 (Figure 9A); in contrast, 27 metabolic pathways were differentially represented at week 4. Alanine, aspartate, glutamate, glycine, serine, and threonine metabolism were increased in group D1, while cysteine and methionine metabolism formed amino acid metabolism in group D2. A higher capacity of streptomycin and phenylpropanoid biosynthesis was also present in group D1. The pyruvate metabolism and citrate cycle (TCA cycle) pathways were increased in Group D2, and the galactose, starch, and sucrose metabolism pathways formed carbohydrate metabolism in Group D1. A higher proportion of nitrogen metabolism pathway involved in energy metabolism characterized group D1, whereas the carbon fixation pathways in prokaryotes were relevant in Group D2. Glycan biosynthesis and metabolism were represented in group D1 by a high proportion of glycosaminoglycan degradation, glycosphingolipid biosynthesis—globo and isoglobo series—and other glycan degradation pathways, while Group D2 presented a high proportion of the peptidoglycan biosynthesis pathway. Relative to lipid metabolism, group D1 showed increased glycerolipid metabolism, fatty acid biosynthesis, and sphingolipid metabolism pathways, while group D1 presented a higher proportion of pathways related to nicotinate and nicotinamide metabolism, cyanoamino acid metabolism, Polyketide sugar unit biosynthesis, and nucleotide metabolism, including the purine metabolism and pyrimidine metabolism pathways. Group D2 had a higher prevalence of metabolic pathways related to the degradation of aromatic compounds and nitrotoluene degradation (Figure 9B).

Thirty-three metabolic pathways with differential prevalence were found at week 12. As for Group D1, 15 out of the 20 routes present at week 4 continued to show a similar pattern; the same was true for four pathways in group D2. Higher abundances of flavone and flavonol biosynthesis, flavonoid biosynthesis; stilbenoid, diarylheptanoid, and gingerol biosynthesis; amino sugar and nucleotide sugar metabolism; one carbon pool by folate and drug metabolism—other enzyme pathways were present in D1, and a high proportion of the arginine and proline metabolism, 2-oxocarboxylic acid metabolism, carbon metabolism, microbial metabolism in diverse environments, porphyrin and chlorophyll metabolism, and biosynthesis of type II polyketide backbone pathways were shown in Group D2 (Figure 9C).

It is interesting to note that a new functional metabolic pathway profile was observed in both groups at week 24. A higher proportion of histidine metabolism; phenylalanine, tyrosine, and tryptophan biosynthesis; citrate cycle (TCA cycle, tricarboxylic acid cycle); glyoxylate and dicarboxylate metabolism; glycerolipid metabolism; carbon fixation pathways in prokaryotes; and oxidative phosphorylation for amino acid, carbohydrate, lipid, and energy metabolism was shown in Group D1. In addition, a high prevalence of the biosynthesis of antibiotics, biosynthesis of secondary metabolites, carbon metabolism, and pantothenate and CoA biosynthesis was observed. In contrast, a high prevalence of pathways related to carbohydrate metabolism, such as amino sugar and nucleotide sugar metabolism, fructose and mannose metabolism, galactose metabolism, glycolysis/gluconeogenesis, pentose phosphate pathway, and starch and sucrose metabolism; a high capacity for streptomycin biosynthesis, sulfur metabolism, peptidoglycan biosynthesis, glycerolipid metabolism, and polyketide sugar unit biosynthesis; and a high ability for xenobiotics biodegradation, including chloroalkane and chloroalkene degradation, naphthalene degradation, and polycyclic aromatic hydrocarbon degradation, were described for Group D2. (Figure 9D).

### 3.5. Metabolic Status and Immune Cell Populations Profiling in VAT

To evaluate the relationship among the changes detected in the GM composition, immune cells phenotype in the VAT, and metabolic markers, we analyzed four mice per group that were randomly sacrificed after 24 weeks post-differential feeding.

**Figure 10:**
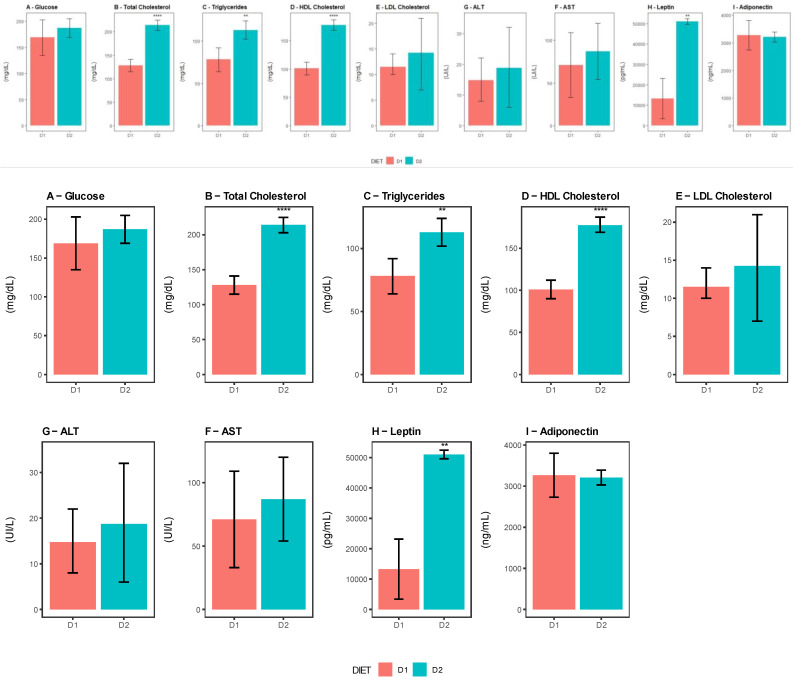
Blood metabolic parameter comparison between groups D1 and D2. (**A**–**I**) Concentrations of blood metabolic parameters, namely glucose, total cholesterol, triglycerides, low-density lipoprotein cholesterol (LDL Cholesterol), high-density lipoprotein cholesterol (HDL Cholesterol), alanine aminotransferase (ALT), aspartate aminotransferase (AST), leptin, and adiponectin. Bar plot representing the parameters evaluated for each group, Group D1 (pink) and Group D2 (sky blue), at week 24 post-starting differential feeding. Data are shown as the mean ± SD of four mice per group. Significant differences were established using the two-tailed Student’s t test **: *p* ≤ 0.01, ****: *p* ≤ 0.0001).

Regarding the metabolic state, it can be seen in Figure 10 that the leptin levels in Group D2 were higher than those in Group D1, whereas the levels of adiponectin did not show significant differences between the groups. Additionally, a significant increase in the total cholesterol values was observed in Group D2 compared with Group D1, which could be attributed mainly to a higher level of HDL-cholesterol particles. The triglyceride levels were also higher in Group D2. No other statistically significant changes were observed in the rest of the parameters evaluated, i.e., glucose, LDL-c, AST, and ALT (Figure 10A–I). Furthermore, Group D2 presented higher body and VAT weights compared with Group D1 (*p*-value = 0.0039 and 0.0007). Supplementary Table S4 shows the statistical comparisons of the metabolic parameters and body and VAT weights.

**Figure 11:**
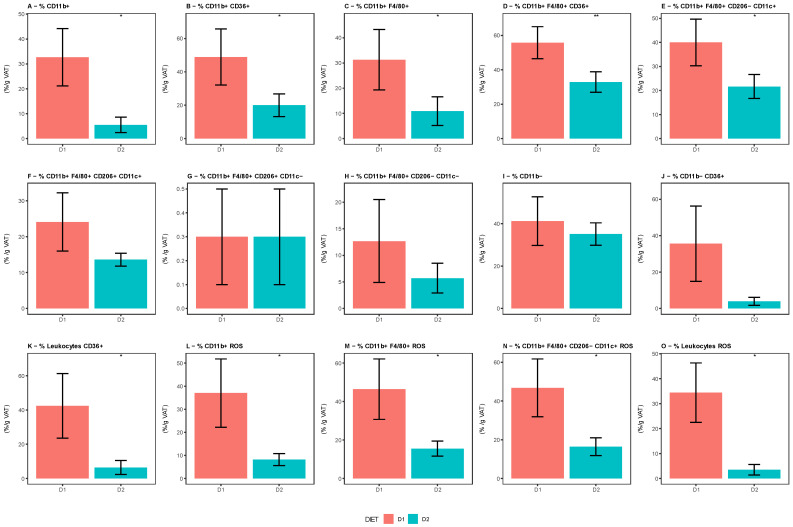
Comparison of immune cell population profiles between Groups D1 and D2. Bar plots representing: (**A**) % CD11b+ cells, (**B**) % CD11b+ CD36+ cells, (**C**) % CD11b+ F4/80+ cells, (**D**) % CD11b+ F4/80+ CD36+ cells, (**E**) % CD11b+ F4/80+ CD206− CD11c+ (M1) cells, (**F**) % CD11b+ F4/80+ CD206+ CD11c+, (**G**) % CD11b+ F4/80+ CD206+ CD11c− (M2) cells, (**H**) % CD11b+ F4/80+ CD206− CD11c− cells, (**I**) % CD11b− cells, (**J**) % CD11b− CD36+ cells, (**K**) % leukocytes CD36+ cells, (**L**) % CD11b+ ROS, (**M**) % CD11b+ F4/80+ ROS, (**N**) % CD11b+ F4/80+ CD206− CD11c+ ROS, and (**O**) % leukocytes ROS evaluated for each group, Group D1 (pink) and Group D2 (sky blue), at week 24 post-starting differential feeding. The results are expressed as the percentage of cells per gram of AT. Data are shown as the mean ± SD of four mice per group. Significant differences were established using the two-tailed Student’s *t*-test (*: *p* ≤ 0.05, **: *p* ≤ 0.01). Abbreviations: VAT: visceral adipose tissue, ROS: reactive oxygen species, CD: cluster of differentiation.

The characterization of immune cells in the VAT showed that mice fed with diet D2 had a significantly lower proportion of cells of myeloid lineage (CD11b+), total macrophages (CD11b+ F4/80+), and pro-inflammatory M1 macrophages (CD11b+ F4/80+ CD206-CD11c+) compared with those fed with diet D1. A marked decrease in ROS production was detected in all of these cells and a similar pattern was found in CD36+ expression, indicating an anti-inflammatory microenvironment in the VAT of group D2. No significant difference in the prevalence of anti-inflammatory M2 macrophages (CD11b+ F4/80+ CD206+ CD11c−) was observed. (Figure 11). Supplementary Table S5 shows the statistical comparison of the cytometry parameters.

**Figure 12:**
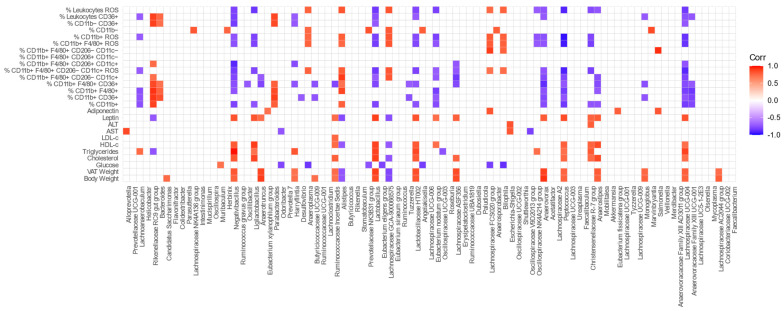
Correlation between body weight, blood metabolic parameters, and immune cells present in VAT with bacterial genera of the gut microbiota. The color is according to the Pearson correlation coefficient distribution; red represents a significant positive correlation, blue represents a significant negative correlation (*p*-value < 0.05), and white represents a non-significant correlation (*p*-value > 0.05). Abbreviations: VAT: visceral adipose tissue, ROS: reactive oxygen species CD: cluster of differentiation, LDL-c: low-density lipoprotein cholesterol, HDL-c: high-density lipoprotein cholesterol, ALT: alanine aminotransferase, AST: aspartate aminotransferase.

In order to determine whether there was a relationship between metabolic parameters, immune cells present in VAT, and the bacterial genera of the GM, we performed a correlation analysis among these attributes. Figure 12 shows the correlation graphs for each of the variables studied.

It can be seen that the abundances of *Lactobacillus* and *Ligilactobacillus* were negatively correlated with pro-inflammatory immune parameters, such as ROS production by total leukocytes and macrophages, M1 macrophages, and CD11b+ cells. Similar results were found for *Anaerostipes*, *Anaerovorax*, *Christensenellaceae R-7 group*, *Eubacterium nodatum group*, *Lachnoanaerobaculum*, *Lactobacillaceae HT002*, *Lachnospiraceae ASF356*, *Lachnospiraceae UCG-004*, *Negativibacillus*, *Oscillospiraceae NK4A214 group*, and *Peptococcus*. In contrast, *Bilophila*, *Anaeroplasma*, *Alistipes*, *Lachnospiraceae GCA-900066575*, and *Lachnospiraceae FCS020 group* were positively correlated with ROS production by immune cells in VAT. Additionally, *Negativibacillus*, *Anaerotruncus*, *Lactobacillus*, *Eubacterium nodatum group*, *Lachnospiraceae ASF356*, *Anaerovorax*, *Peptococcus*, *Anaerostipes*, and *Lachnospiraceae UCG-004* were negatively correlated with the prevalence of pro-inflammatory M1 macrophages in VAT, while *Rikenellaceae RC9 gut group*, *Alistipes*, and *Lachnospiraceae GCA-900066575* were positively correlated.

The abundances of *Alistipes* and *Lachnospiraceae GCA-900066575* were correlated inversely with the mice body and VAT weights. Moreover, these genera plus *Rikenellaceae RC9 Gut Group* were negatively correlated with the leptin levels. In contrast, the abundances of *Butyricicoccaceae UCG-009*, *Candidatus Saccharimonas*, *Harryflintia*, *Lachnospiraceae AC2044 group*, *Lachnospiraceae ASF356*, *Lachnospiraceae UCG-004*, *Lactobacillaceae HT002*, *Lactobacillus*, *Monoglobus*, *Negativibacillus*, *Peptococcus*, and *Ruminococcaceae Incertae Sedis* were positively correlated with the body weight and leptin levels.

*Anaerostipes*, *Christensenellaceae R-7 group*, *Eubacterium nodatum group*, *Harryflintia*, *Lachnoanaerobaculum*, *Lachnospiraceae ASF356*, *Lachnospiraceae UCG-004*, *Lactobacillaceae HT002*, *Lactobacillus*, *Ligilactobacillus*, *Negativibacillus*, and *Oscillospiraceae NK4A214 group* were positively correlated with some of the lipid levels studied (TC, HDL-c or TG). Only *Ruminococcaceae Incertae Sedis* was correlated with LDL-c.

*Odoribacter*, *Anaeroplasma*, *Prevotellaceae NK3B31 group*, *Eubacterium eligens group*, *Angelakisella*, *Bilophila*, and *Lachnospiraceae FCS020 group* showed a significant negative correlation with the glucose levels, unlike the genus *Muribaculum*, which showed a positive correlation. Supplementary Table S6 presents the correlation coefficient for variables showing significant correlations.

## 4. Discussion

There is increasing evidence that the GM plays a fundamental role in the regulation of homeostatic mechanisms and metabolism to exert either beneficial or detrimental effects on the host’s health. Different studies have shown that the qualitative and quantitative composition of the diet is a key factor modulating the host microbiota structure and function, or leading to gut dysbiosis, which may impact the development or prevention of certain NCDs [18]. Indeed, diet influences the composition of the microbiota, providing nutrients for both the host and the gut bacteria [44]. In this context, the aim of this study was to analyze the effect of the diet’s composition on the development of obesity, GM structure, and immune metabolic response on VAT, focusing on the fatty acids and omega-3 content.

We observed that, after a feeding period of 12 and 24 weeks, mice fed with diet D2 showed a significant increase in body weight and obesity. Remarkably, these results reveal that it is feasible to induce obesity with a considerably lower fat content than that traditionally used in HFD models, generating an experimental model and changes that better reflect the biological conditions leading to human obesity [22]. Besides contributing to the development of obesity through the first 12 weeks, diet D2 generated significant changes in the GM composition, resulting in an increase in *Firmicutes* and a decrease in *Bacteroidota*, which is in agreement with findings reported previously [7,13,14,45,46]. In addition, other differences previously reported, such as increases in *Campylobacterota* [47], *Cyanobacteria* [48], *Deferribacterota*, and *Desulfobacterota* [49,50], were evidenced. These taxa are considered harmful or potentially pathogenic, as they favor the development of inflammation and alter the intestinal microenvironment, generating changes in intestinal permeability, enabling, in turn, the development of metabolic endotoxemia [49,51,52]. When analyzing, at the genus level, the bacteria present, we observed that group D2 showed a greater abundance of taxa involved in fiber digestion contributing to the production of SCFAs, including *Eubacterium eligens group***,** *Eubacterium nodatum group***,** *Lachnospiraceae AC2044 group***,** *Prevotellaceae group NK3B31***,** *Ruminococcaceae UBA1819*, among others. Furthermore, we found that mice with a higher dietary fat intake had higher abundances of *Oscillibacter***,** *Harryflintia***,** *Mucispirillum***,** *Flavonifractor*, and *Anaerotruncus*. These bacteria have been related to inflammatory processes and the development of different chronic diseases in numerous reports. Lam et al. reported a significant increase in the abundance of *Oscillibacter* in HFD-fed mice compared with LFD-fed mice. At the same time, these changes were associated with weight gain, as well as being negatively correlated with gut permeability [53]. Similar results have been demonstrated by Jo et al., who also reported an increase in *Harryflintia* under a HFD [54]. On the other hand, an increase in *Mucispirillum*, a potentially pathogenic genus, has been reported in different conditions associated with gut inflammation [55] and related to HFD [56]. Additionally, an increase in the abundance of *Flavonifractor* and a decrease in the *Christensenellaceae* family have been associated with the development of affective disorders in humans; these findings are also associated with greater oxidative stress and low-grade systemic inflammation [57]. *Anaerotruncus*, a butyrate producer, exhibited higher abundance in mice fed with diet D2; to note, this genus has also been associated with the development of obesity [58]. Overall, these findings could explain, at least in part, some of the potential mechanisms of obesity-associated dysbiosis, which involve increased ability to harvest and store energy from dietary components, mainly exacerbated ability to produce SCFA [13], leading to increased intestinal permeability and inflammation in this experimental model, as evidenced by the presence of a cellular infiltrate rich in pro-inflammatory M1 macrophages and a low number of anti-inflammatory M2 macrophages in the VAT. Overall, these findings show that most of the changes produced in the GM by this medium-fat-content diet are compatible with the previously described obesity-associated dysbiosis.

With regard to mice fed with diet D1, they showed a dominance of mainly SCFA-producing taxa and lactic acid producers, both considered beneficial bacteria. Surprisingly, a higher prevalence of *Bilophila* and *Desulfovibrio* genera was also observed, contrary to previous reports, where levels of *Bilophila wadsworthia*, a sulfite-reducing pathobiont associated with increased intestinal inflammation, were found when mice were fed a diet enriched with milk fat [59]. Similar results were presented by Shen et al., where mice fed with HFD (60% of energy from fat; 95% lard and 5% soybean oil) had a greater abundance of three types of sulfidogenic bacteria (*Desulfobacter* spp., *Desulfovibrio* spp., and *Bilophila wadsworthia*) in colonic mucosa compared with mice fed with LFD (10% of energy from fat; 55% soybean oil and 45% lard) after 20 weeks of feeding [20]. It has been postulated that sulfidogenic bacteria can contribute to diminished epithelial integrity and increased intestinal permeability, possibly through the production of the pro-inflammatory and genotoxic gas hydrogen sulfide [20]. In contrast, a higher prevalence of the genus *Muribaculum*, associated with intestinal barrier function markers, was also detected [60].

Interestingly, at week 24, the changes observed in the composition of the GM in Group D2 were different to the alterations described earlier. In line with different reports, these findings may be attributable to the sustained dietary intake of omega-3; we detected a correction of the *Firmicutes* and *Bacteroidota* abundances, although the differences between groups D1 and D2 were not statistically significant. Su et al. showed that the contribution of high doses of PUFAs, especially EPAs and DHAs, reversed the changes produced by HFD in the abundance of *Firmicutes* and *Bacteroidota* within a period of 18 weeks [61]. Additionally, mice fed with D2 presented an increase in the abundances of certain bacterial genera, such as *Lachnospiraceae AC2044 group*, *Lachnospiraceae ASF356*, *Lachnospiraceae UCG-003*, and *Lachnospiraceae UCG-004*, which are related to the production of SCFAs, and a decrease in the abundance of *Faecalibacterium*. In our cohort, we also observed an enrichment of *Anaerostipes*, one of the most abundant butyrogenic bacteria of the healthy microbiota and a high consumer of lactate in the colon [44,62]. Remarkably, we found greater abundances of *Akkermansia* and *Lactobacillus* compared with the GM from mice fed with D1; the former has the ability to improve the intestinal microenvironment, increasing the thickness and maintaining the barrier function of the intestinal mucosa [63]. Additionally, it has been associated with weight loss by controlling the expression of genes related to fat metabolism [64]. This is in agreement with a report by Watson et al., demonstrating that the administration of omega-3 PUFAs supported an increase in the abundances of the *Clostridiaceae*, *Sutterellaceae*, and *Akkermansiaceae* families, among other changes that were reversible after a washout period [65]. Similar results were found when examining the abundance of *Lactobacillus*, which is known to decrease intestinal inflammation and provide competitive resistance against pathogens [65,66].

The different impact of diet over time observed in our study is probably due to the fact that the proportion of the saturated and/or omega-6 fatty acid component of D2 would favor the growth of bacteria associated with the development of metabolic endotoxemia throughout the first 12 weeks of differential feeding, favoring the development of obesity and potential associated metabolic disturbances. Later, throughout weeks 12 and 24, the effect of omega-3 fatty acids became predominant, favoring the growth of bacteria with probiotic potential, which would contribute to controlling the intestinal inflammation, modulating both metabolism and the immune system, as observed by the presence of an anti-inflammatory environment in VAT [57,61]. Further studies to confirm these considerations are anticipated. Interestingly, the genera *Harryflintia*, *Lachnospiraceae AC2044 group*, *Lachnospiraceae ASF356*, *Mucispirillum*, *Ruminococcaceae UBA1819,* and *Tyzzerella* could be considered a microbial signature for GM in mice fed with a fatty-acid-rich diet, as they presented the same distribution profile in the differential analysis between diets over time.

Our study also explored the metabolic activities of GM from Groups D1 and D2; the differential feeding generated distinct functional profiles in each group at different times, indicating that the content of dietary fatty acids influenced the functional profile of the microbiota in a time-dependent manner. However, knowledge of the connections between omega-3 and the GM metabolic activities is still limited [44]. Even so, when evaluating the influence of an omega-3-supplemented fat diet, we found interesting effects on metabolic parameters and on the modulation of the immunological microenvironment in VAT. It is very well known that the accumulation of AT in obesity is characterized by changes in the circulating levels of various adipokines, such as leptin and adiponectin [67]. Leptin affects both endocrine functions and different aspects of the immune response; it has been demonstrated that elevated leptin in obesity contributes to a low-grade inflammatory state [68], acting on the regulation of T cells, macrophages, and increasing the production of pro-inflammatory cytokines and ROS [68,69,70]. In contrast, adiponectin, an anti-inflammatory adipokine, decreased in obesity, acts as an insulin-sensitizing hormone in muscles and the liver; low levels of adiponectin contribute to peripheral insulin resistance [71]. Our results showed high levels of leptin at week 24; however, the comparable levels of adiponectin in Groups D1 and D2 could explain the significant increase in body weight in Group D2, almost double that of their counterpart, without the development of metabolic alterations for carbohydrates or for the liver enzymes AST and ALT. In agreement with these results, Chacińska et al. demonstrated that PUFAs can prevent the development of insulin resistance in response to high-fat feeding and can regulate the expression and secretion of adipocytokines in animal models [72]. Finally, the considerable increase in HDL-c levels in Group D2 was attributable to the intake of omega-3, as described by different authors both in humans and animal models [73,74,75,76].

Although obesity is characterized by the inflammation of AT, with an important infiltrate of mainly pro-inflammatory M1 macrophages [5,6], our findings show that the VAT of mice fed with diet D2 presented lower numbers of myeloid cells, lower total and M1 macrophages infiltration, and decreased production of ROS compared with mice fed the control diet. No differences in the percentage of anti-inflammatory M2 macrophages were found. Overall, these results show that omega-3 generates an environment with anti-inflammatory characteristics in the VAT, maintaining its architecture and functionality, which is reflected by the adiponectin levels found. This is in agreement with previous reports [77]. Finally, we were able to corroborate that the abundance of certain bacterial genera in the GM was significantly correlated not only with the presence of different cell populations in the VAT, but also with a series of metabolic parameters. It is interesting to remark here that the most outstanding finding of this analysis is the negative correlation found between the abundance of *Lactobacillus* and *Ligilactobacillus* with the production of ROS by total leukocytes, CD11b+ cells, and M1 macrophages. It is important to note that these genera presented differential abundances at week 24 after feeding D2, showing that the effect of long-term intake of omega-3 on the modulation of the intestinal microbiota favored immunomodulation in VAT. In this context, Huang et al. demonstrated that *Lactobacillus*-induced anti-inflammatory macrophages associated with REG3γ in the intestinal lamina propria may play a role in adipose tissue homeostasis and be involved in high-fat-diet-mediated resistance to obesity [78]. Regarding the *Christensenellaceae* family, which also increased in Group D2, it has been shown that certain species have powerful immunomodulatory properties [79], correlating negatively with ROS production by immune cells in VAT, as well as intestinal inflammation [79,80], although further studies should be performed to confirm these results. On the other hand, *Alistipes* and *Lachnospiraceae GCA-900066575* showed an inverse correlation with the mice body and VAT weight. This has been also reported in humans by Tavella et al., who stated that a high abundance of *Alistipes* was associated with a lower proportion of VAT and healthier metabolic profile in older adults [80]. Additionally, Pesoa et al. showed that the abundance of certain species of the genus *Alistipes*, such as *Alistipes shahii* and *Alistipes* sp., was significantly lower in obese subjects in comparison to normal-weight subjects [81]. In contrast, other genera associated with metabolic endotoxemia or the high production of SCFA were correlated positively with body weight, as has been demonstrated by others [13,54]. Additionally, different findings suggest that the GM has the capacity to modulate the blood lipid composition through distinct mechanisms, such as microbial products or bile acid metabolism regulation [82,83,84]. We found that *Rikenellaceae RC9 Gut Group* and *Oscillospiraceae UCG-003* were correlated inversely with the triglyceride level, which is in agreement with previous findings [85]. Moreover, we observed that *Lactobacillus* was correlated positively with the TC, TG, and HDL-c levels, which is in opposition to reports where mice treated with probiotics, *Lactobacillus curvatus* alone or together with *Lactobacillus plantarum*, fed with a HFD showed reduced cholesterol in the plasma and liver [86]. In addition, *Lachnospiraceae UCG-004* also showed a positive correlation with HDL-c, similar to results reported by Lan et al. in the Chinese population [87].

Taken together, these results suggest that the administration of a diet with medium amounts of saturated fat supplemented with omega-3 fatty acids contributed to the development of obesity, associated with specific changes in the composition and diversity of the intestinal microbiota, metabolic pathways profiles, and VAT immuno-metabolism in a time-dependent manner. The stabilization of weight gain with preserved metabolic state noted by week 24 after differential feeding was probably due to beneficial changes in the intestinal microbial communities, such as the increased abundance of probiotic bacteria, such as *Lactobacillus* and *Ligilactobacillus*, promoted by the sustained intake of omega-3 PUFAs, which, in turn, contributed to the generation of an anti-inflammatory microenvironment in the VAT with a marked decrease in pro-inflammatory cells and ROS production. Despite these findings, our study has some limitations: the sample size to determine the microbiota composition, metabolic markers, and immune cell profile was small (*n* = 8 and *n* = 4), respectively. On the other hand, we only analyzed the bacterial taxonomy up to the genus level; thus, it was possible that information on the existence of some species/strains that could be considered microbial biomarkers associated with the contribution of omega-3 is missing. The results obtained do not allow us to determine the mechanism underlying the relationships among nutrition, GM, and immune-metabolism. These observations need further validation by performing studies of metabolic parameters and immunological profiles in VAT at different time intervals and considering a diet of medium fat composition without omega-3 supplements.

## 5. Conclusions

Remarkably, these results reveal that it is feasible to induce obesity with a considerably lower fat content than that traditionally used in HFD models, generating an experimental model and changes that better reflect biological conditions contributing to human obesity. The constant and sustained administration of omega-3 fatty acids induced specific changes in the GM composition, mainly with increases in *Lactobacillus and Ligilactobacillus* species, which, in turn, modulated the immune metabolic response of AT in this mouse model of obesity.

The identification of different taxonomic signatures associated with the healthy functionality of VAT and metabolic state in obesity could be a new, promising tool to design treatment or prevention strategies for obesity and associated comorbidities, focusing on the modulation of the intestinal microbiota through the consumption of omega-3 fatty acids.

## Data Availability

Sequencing data are accessible in the National Center for Bio-technology Information (NCBI) database under BioProject accession number PRJNA929200 (https://ncbi.nlm.nih.gov/bioproject/?term=PRJNA929200).

## Supplementary Materials

The following supporting information can be downloaded at: https://www.mdpi.com/article/10.3390/nu15061404/s1, Supplementary Table S1A: Alpha Diversity Index by Time; Supplementary Table S1B: Alpha Diversity Index by Diet; Supplementary Table S1C: Post hoc analysis for significant Friedman test; Supplementary Table S2: Relative abundance comparison at the phylum level; Supplementary Table S3: Relative abundance comparison at the genus level: Supplementary Table S4: Blood metabolic parameters comparison; Supplementary Table S5: Immune cells population in VAT comparison; Supplementary Table S6: Variables showing significant correlations.
