# Supplementary material for: Omega-3-Supplemented Fat Diet Drives Immune Metabolic Response in Visceral Adipose Tissue by Modulating Gut Microbiota in a Mouse Model of Obesity
Source: Nutrients. 2023 Mar 15;15(6):1404. doi: 10.3390/nu15061404 (PMC10054794; doi:10.3390/nu15061404)
Supplement: Supplementary file 1 [file nutrients-15-01404-s001.zip › nutrients-2223128-supplementary.pdf]

Supplementary Materials

Supplementary Table S1A: Alpha Diversity Index by Time

Supplementary Table S1A: *Alpha Diversity Index by Time*

	Week 0						
	DIET 1			DIET 2			
	Median	***Min***	***Max***	***Median***	***Min***	***Max***	**p-value**
Observed ASVs	931	477	1043	944.5	883	998	0.5737
Shannon Index	5.2910	4.6935	5.6325	5.4378	5.2185	5.7197	0.1605
Simpson Index	0.9855	0.9819	0.9915	0.9864	0.9827	0.9934	0.1949
	**Week 4**						
	**DIET 1**			**DIET 2**			
	Median	***Min***	***Max***	***Median***	***Min***	***Max***	**p-value**
Observed ASVs	947	840	1042	1116.5	1075	1173	**0.0002**
Shannon Index	5.3287	4.7586	5.5727	5.8203	5.6461	5.9144	**0.0002**
Simpson Index	0.9858	0.9538	0.9893	0.9932	0.9907	0.9942	**0.0002**
	**Week 12**						
	**DIET 1**			**DIET 2**			
	Median	***Min***	***Max***	***Median***	***Min***	***Max***	**p-value**
Observed ASVs	1035	958	1104	1107	1014	1157	**0.0207**
Shannon Index	5.4457	5.3138	5.6586	5.8946	5.7021	5.9963	**0.0002**
Simpson Index	0.9869	0.9826	0.9914	0.9940	0.9915	0.9947	**0.0002**
	**Week 24**						
	**DIET 1**			**DIET 2**			
	Median	***Min***	***Max***	***Median***	***Min***	***Max***	**p-value**
Observed ASVs	995	859	1133	1080	1044	1177	**0.0148**
Shannon Index	5.5636	5.3234	6.0780	5.7755	5.6106	6.0150	0.0830
Simpson Index	0.9904	0.9866	0.9954	0.9927	0.9910	0.9948	**0.0499**

Supplementary Table S1A: Alpha diversity index (Observed ASVs, Shannon and Simpson Index) for groups D1 and D2 at different time feeding. Significant differences established using the Wilcoxon signed-rank test ($p<0.05$). p-values in bold style were statistically significant.

Supplementary Table S1B: Alpha Diversity Index by Diets

Supplementary Table S1B: Alpha Diversity Index by Diets

	DIET1				DIET2			
	**Week 0**	**Week 4**	**Week 12**	**Week 24**	**Week 0**	**Week 4**	**Week 12**	**Week 24**
Observed ASVs - Median	931	947	1035	995	945	1117	1107	1080
Observed ASVs - Min	477	840	958	859	883	1075	1014	1044
Observed ASVs - Max	1043	1042	1104	1133	998	1173	1157	1177
p - value	0.1357				**0.0024**			
Shannon Index - Median	5.2910	5.3287	5.4457	5.5636	5.4378	5.8203	5.8946	5.7755
Shannon Index - Min	4.6935	4.7586	5.3138	5.3234	5.2185	5.6461	5.7021	5.6106
Shannon Index - Max	5.6325	5.5727	5.6586	6.0780	5.7197	5.9144	5.9963	6.0150
p - value	**0.0336**				**0.0022**			
Simpson Index - Median	0.9855	0.9858	0.9869	0.9904	0.9864	0.9932	0.994	0.9927
Simpson Index - Min	0.98194	0.95383	0.98265	0.98658	0.9827	0.9907	0.9915	0.9910
Simpson Index - Max	0.99152	0.98933	0.99141	0.99537	0.9934	0.9942	0.9947	0.9948
p - value	**0.05**				**0.0239**			

Supplementary Table S1B: Alpha diversity index (Observed ASVs, Shannon and Simpson Index) for week 0, 4, 12 and 24 in the different diets. Significant differences established using the Friedman test ($p<0.05$). p-values in bold style were statistically significant.

Supplementary Table S1C: Post hoc analysis for significant Friedman test

Supplementary Table S1C: Post hoc analysis for significant Friedman test

	DIET 1			DIET 2		
Comparison	**Observed ASVs p-value adj**	**Shannon p-value adj**	**Simpson p-value adj**	**Observed ASVs p-value adj**	**Shannon p-value adj**	**Simpson p-value adj**
Week 0 - Week 4	-	1.000	1.000	**0.047**	0.094	0.328
Week 0 - Week 12	-	0.888	1.000	**0.047**	**0.047**	**0.047**
Week 0 - Week 24	-	0.654	0.328	**0.047**	**0.047**	0.654
Week 4 - Week 12	-	0.328	1.000	1.000	1.000	1.000
Week 4 - Week 24	-	**0.047**	**0.047**	1.000	1.000	1.000
Week 12 - Week 24	-	1.000	0.328	1.000	0.328	0.888

Supplementary Table S1C: Post hoc analysis for Alpha diversity index (Observed ASVs, Shannon and Simpson Index) with significant Friedman test for week 0, 4, 12 and 24 in the different diets. Significant differences established using the Wilcoxon signed-rank test ($p<0.05$). p-values in bold style were statistically significant.

Supplementary Table S2: Relative abundance comparison at Phylum level

Supplementary Table 2: *Relative abundance comparison at Phylum level*

	Week 0						
		DIET1			DIET2		
	**Median**	**Min**	**Max**	**Median**	**Min**	**Max**	***p-value***
Actinobacteriota	0.0002	0.0000	0.0008	0.0000	0.0000	0.0001	0.0716
Bacteroidota	0.6699	0.5166	0.7285	0.6095	0.2582	0.7542	0.4948
Campylobacterota	0.0116	0.0039	0.0225	0.0234	0.0044	0.0380	0.1893
Cyanobacteria	0.0000	0.0000	0.0016	0.0001	0.0000	0.0007	0.8663
Deferribacterota	0.0025	0.0007	0.0089	0.0033	0.0008	0.0137	0.4942
Desulfobacterota	0.0058	0.0035	0.0131	0.0085	0.0047	0.0205	0.1715
Firmicutes	0.2196	0.1187	0.3808	0.2954	0.1348	0.6280	0.3720
Patescibacteria	0.0048	0.0001	0.0103	0.0061	0.0025	0.0128	0.4005
Proteobacteria	0.0831	0.0619	0.1505	0.0609	0.0417	0.0921	0.1893
Verrucomicrobiota	0.0000	0.0000	0.0009	0.0000	0.0000	0.0000	0.3816
	**Week 4**						
		**DIET1**			**DIET2**		
	**Median**	**Min**	**Max**	**Median**	**Min**	**Max**	***p-value***
Actinobacteriota	0.0001	0.0000	0.0007	0.0001	0.0000	0.0002	0.3575
Bacteroidota	0.6721	0.6152	0.7727	0.5529	0.4621	0.6716	**0.0074**
Campylobacterota	0.0129	0.0073	0.0270	0.0272	0.0107	0.0406	**0.0156**
Cyanobacteria	0.0000	0.0000	0.0002	0.0003	0.0001	0.0008	**0.0099**
Deferribacterota	0.0022	0.0001	0.0041	0.0127	0.0047	0.0222	**0.0009**
Desulfobacterota	0.0059	0.0020	0.0075	0.0207	0.0143	0.0353	**0.0009**
Firmicutes	0.2002	0.1173	0.2568	0.2924	0.2090	0.3813	**0.0054**
Patescibacteria	0.0126	0.0101	0.0241	0.0218	0.0072	0.0399	0.0831
Proteobacteria	0.0832	0.0718	0.1027	0.0617	0.0470	0.0735	**0.0019**
Verrucomicrobiota	0.0000	0.0000	0.0012	0.0001	0.0000	0.0006	0.2304
	**Week 12**						
		**DIET1**			**DIET2**		
	**Median**	**Min**	**Max**	**Median**	**Min**	**Max**	***p-value***
Actinobacteriota	0.0001	0.0000	0.0005	0.0000	0.0000	0.0001	**0.0043**
Bacteroidota	0.6603	0.6124	0.7147	0.5376	0.4350	0.6003	**0.0009**
Campylobacterota	0.0124	0.0050	0.0159	0.0179	0.0086	0.0352	0.0831
Cyanobacteria	0.0001	0.0000	0.0007	0.0002	0.0000	0.0012	0.3071
Deferribacterota	0.0015	0.0004	0.0047	0.0092	0.0033	0.0115	**0.0019**
Desulfobacterota	0.0058	0.0023	0.0110	0.0332	0.0214	0.0412	**0.0009**
Firmicutes	0.2157	0.1551	0.2395	0.3238	0.2568	0.4184	**0.0009**
Patescibacteria	0.0129	0.0119	0.0239	0.0146	0.0011	0.0185	0.8748
Proteobacteria	0.0967	0.0508	0.1428	0.0612	0.0520	0.0808	**0.0181**
Verrucomicrobiota	0.0002	0.0000	0.0011	0.0000	0.0000	0.0002	0.0823
	**Week 24**						
		**DIET1**			**DIET2**		
	**Median**	**Min**	**Max**	**Median**	**Min**	**Max**	***p-value***

Actinobacteriota	0.0002	0.0000	0.0004	0.0000	0.0000	0.0002	0.1524
Bacteroidota	0.6711	0.4880	0.7742	0.5430	0.4993	0.6844	0.2271
Campylobacterota	0.0159	0.0069	0.0256	0.0107	0.0020	0.0218	0.1893
Cyanobacteria	0.0005	0.0000	0.0010	0.0001	0.0000	0.0002	**0.0127**
Deferribacterota	0.0019	0.0002	0.0050	0.0045	0.0001	0.0082	0.2701
Desulfobacterota	0.0054	0.0010	0.0139	0.0163	0.0083	0.0311	**0.0039**
Firmicutes	0.1966	0.1298	0.4149	0.3025	0.2042	0.3722	0.1563
Patescibacteria	0.0149	0.0061	0.0200	0.0235	0.0159	0.0688	**0.0100**
Proteobacteria	0.0696	0.0413	0.1085	0.0763	0.0617	0.0879	0.5635
Verrucomicrobiota	0.0003	0.0000	0.0021	0.0000	0.0000	0.0010	0.3253

Supplementary Table S2: Relative abundance comparison between diet groups at Phylum level in the different weeks of study. Significant differences established using the Wilcoxon signed-rank test ($p<0.05$). p-values in bold style were statistically significant.

Supplementary Table S3: Relative abundance comparison at Genus level

Supplementary Table S3: *Relative abundance comparison at Genus level*

	Week 0						
	DIET1			DIET2			
	**Median**	**Min**	**Max**	**Median**	**Min**	**Max**	**p-value**
Alloprevotella	0.1255	0.0311	0.1729	0.1140	0.0069	0.1544	0.4948
Lachnoanaerobaculum	0.0185	0.0001	0.0636	0.0209	0.0114	0.0478	0.7929
Helicobacter	0.0243	0.0111	0.0609	0.0441	0.0150	0.0660	0.1278
Rikenellaceae RC9 gut group	0.0205	0.0090	0.0486	0.0333	0.0061	0.0649	0.6365
Candidatus Saccharimonas	0.0130	0.0001	0.0271	0.0121	0.0084	0.0381	0.7132
Bacteroides	0.1537	0.0947	0.5134	0.1527	0.0790	0.2466	1.0000
Prevotellaceae UCG-001	0.0759	0.0030	0.1472	0.0748	0.0251	0.1799	0.7132
Flavonifractor	0.0079	0.0008	0.0120	0.0082	0.0055	0.0124	0.8748
Colidextribacter	0.0221	0.0034	0.0376	0.0253	0.0140	0.0320	0.7132
Parasutterella	0.0271	0.0088	0.0787	0.0133	0.0005	0.0443	0.0520
Odoribacter	0.0193	0.0000	0.0658	0.0290	0.0181	0.0690	0.1893
Lachnospiraceae NK4A136 group	0.1739	0.0040	0.3049	0.1499	0.1063	0.3421	0.9581
Intestinimonas	0.0108	0.0008	0.0169	0.0107	0.0077	0.0147	1.0000
Mucispirillum	0.0090	0.0020	0.0168	0.0084	0.0027	0.0215	0.9581
Oscillospira	0.0031	0.0001	0.0067	0.0025	0.0017	0.0038	0.5635
Muribaculum	0.0285	0.0079	0.0914	0.0262	0.0004	0.0680	0.7132
Herbinix	0.0129	0.0008	0.0400	0.0125	0.0073	0.0460	0.3184
Negativibacillus	0.0052	0.0017	0.0089	0.0055	0.0018	0.0104	0.7132
Parabacteroides	0.0238	0.0040	0.0407	0.0120	0.0001	0.0195	0.0831
Alistipes	0.0207	0.0029	0.0497	0.0186	0.0137	0.1007	0.7132
Ruminococcus gnavus group	0.0048	0.0005	0.0067	0.0052	0.0018	0.0121	0.4309
Oscillibacter	0.0069	0.0002	0.0107	0.0090	0.0040	0.0186	0.0831
Ligilactobacillus	0.0046	0.0017	0.0224	0.0055	0.0007	0.0109	0.7929
Anaerotruncus	0.0008	0.0000	0.0060	0.0010	0.0000	0.0085	0.8335
Eubacterium xylanophilum group	0.0040	0.0001	0.0138	0.0038	0.0020	0.0100	0.8748
Prevotella 7	0.0014	0.0002	0.0045	0.0007	0.0002	0.0038	0.7132
Harryflintia	0.0016	0.0001	0.0034	0.0027	0.0006	0.0044	0.1563
Desulfovibrio	0.0023	0.0002	0.0039	0.0031	0.0011	0.0036	0.4948
Tuzzerella	0.0031	0.0000	0.0060	0.0041	0.0008	0.0061	0.4309
Anaeroplasma	0.0028	0.0000	0.0128	0.0046	0.0016	0.0208	0.0831
Butyricicoccaceae UCG-009	0.0020	0.0000	0.0063	0.0030	0.0005	0.0036	0.3184
Erysipelatoclostridium	0.0009	0.0003	0.1317	0.0002	0.0000	0.0027	0.0831
Ruminococcaceae UCG-001	0.0004	0.0000	0.0043	0.0000	0.0000	0.0014	0.2620
Lachnoclostridium	0.0064	0.0021	0.0111	0.0095	0.0050	0.0238	0.1893
Ruminococcaceae Incertae Sedis	0.0024	0.0006	0.0051	0.0033	0.0014	0.0054	0.3184
Butyricicoccus	0.0021	0.0002	0.0078	0.0016	0.0000	0.0028	0.4309
Stomatobaculum	0.0002	0.0000	0.0053	0.0003	0.0000	0.0006	0.9575
Prevotellaceae NK3B31 group	0.0000	0.0000	0.0029	0.0000	0.0000	0.0004	1.0000
Lactobacillus	0.0005	0.0000	0.0163	0.0001	0.0000	0.0010	0.1179
Eubacterium eligens group	0.0002	0.0000	0.0019	0.0019	0.0004	0.0060	**0.0136**
Lachnospiraceae GCA-900066575	0.0109	0.0018	0.0229	0.0065	0.0015	0.0189	0.4309
Rikenella	0.0032	0.0000	0.0142	0.0042	0.0008	0.0152	0.6365

Ruminococcus	0.0005	0.0000	0.0058	0.0000	0.0000	0.0032	0.2976
Acetatifactor	0.0032	0.0000	0.0144	0.0065	0.0008	0.0152	0.5635
Lactobacillaceae HT002	0.0000	0.0000	0.0084	0.0000	0.0000	0.0002	0.8360
Angelakisella	0.0007	0.0001	0.0022	0.0006	0.0002	0.0013	0.7132
Lachnospiraceae ASF356	0.0038	0.0009	0.0202	0.0051	0.0000	0.0108	0.8748
Lachnospiraceae UCG-006	0.0029	0.0004	0.0041	0.0016	0.0009	0.0087	0.9581
Oscillospiraceae UCG-003	0.0005	0.0000	0.0016	0.0004	0.0000	0.0020	0.8745
Eubacterium siraeum group	0.0011	0.0000	0.0220	0.0027	0.0005	0.0096	0.5635
Ruminococcaceae UBA1819	0.0000	0.0000	0.0003	0.0000	0.0000	0.0000	0.1709
Dubosiella	0.0009	0.0000	0.0166	0.0003	0.0000	0.0015	0.2445
Paludicola	0.0013	0.0000	0.0024	0.0006	0.0001	0.0016	0.0661
Lachnospiraceae FCS020 group	0.0008	0.0001	0.0026	0.0015	0.0004	0.0117	0.1893
Anaerosporobacter	0.0007	0.0000	0.0055	0.0000	0.0000	0.0012	0.1389
Bilophila	0.0002	0.0000	0.0007	0.0004	0.0000	0.0007	0.3973
Lachnospiraceae UCG-001	0.0008	0.0000	0.0046	0.0007	0.0002	0.0080	0.7130
Escherichia-Shigella	0.0008	0.0004	0.0057	0.0000	0.0000	0.0002	0.0007
Oscillospiraceae UCG-002	0.0002	0.0000	0.0005	0.0004	0.0000	0.0009	0.1550
Shuttleworthia	0.0007	0.0000	0.0043	0.0017	0.0000	0.0041	0.7923
Eubacterium nodatum group	0.0017	0.0002	0.0044	0.0023	0.0010	0.0032	0.5635
Oscillospiraceae V9D2013 group	0.0003	0.0000	0.0009	0.0004	0.0000	0.0008	0.8335
Oscillospiraceae NK4A214 group	0.0004	0.0000	0.0007	0.0003	0.0000	0.0010	1.0000
Anaerovorax	0.0000	0.0000	0.0007	0.0000	0.0000	0.0007	1.0000
Roseburia	0.0017	0.0002	0.0070	0.0027	0.0008	0.0092	0.2271
Lachnospiraceae UCG-003	0.0000	0.0000	0.0024	0.0001	0.0000	0.0068	0.5009
Ureaplasma	0.0005	0.0000	0.0053	0.0015	0.0000	0.0112	0.3649
Faecalibaculum	0.0000	0.0000	0.0089	0.0000	0.0000	0.0000	0.0764
Christensenellaceae R-7 group	0.0004	0.0000	0.0020	0.0002	0.0000	0.0008	0.3973
Anaerostipes	0.0001	0.0000	0.0004	0.0001	0.0000	0.0005	0.4761
Mobilitalea	0.0005	0.0001	0.0022	0.0006	0.0003	0.0014	0.5635
Lachnospiraceae A2	0.0005	0.0003	0.0016	0.0013	0.0002	0.0052	0.1278
Akkermansia	0.0000	0.0000	0.0036	0.0000	0.0000	0.0000	0.3816
Peptococcus	0.0008	0.0000	0.0021	0.0011	0.0007	0.0018	0.5635
Eubacterium fissicatena group	0.0005	0.0000	0.0016	0.0007	0.0000	0.0014	1.0000
Tyzzerella	0.0004	0.0000	0.0033	0.0000	0.0000	0.0007	0.1480
Lachnospiraceae UCG-009	0.0003	0.0000	0.0031	0.0001	0.0000	0.0011	0.3514
Monoglobus	0.0004	0.0000	0.0022	0.0001	0.0000	0.0005	0.2497
Anaerovoracaceae Family XIII UCG-001	0.0002	0.0000	0.0006	0.0001	0.0000	0.0005	0.5465
Marvinbryantia	0.0003	0.0000	0.0018	0.0001	0.0000	0.0005	0.2202
Salmonella	0.0000	0.0000	0.0020	0.0000	0.0000	0.0001	0.2697
Lactococcus	0.0008	0.0000	0.0041	0.0001	0.0000	0.0006	0.0569
Anaerovoracaceae Family XIII AD3011 group	0.0001	0.0000	0.0012	0.0001	0.0000	0.0009	0.9553
Lachnospiraceae UCG-004	0.0003	0.0000	0.0009	0.0003	0.0000	0.0011	0.7509
Veillonella	0.0002	0.0000	0.0004	0.0000	0.0000	0.0004	0.1480
Merdibacter	0.0000	0.0000	0.0003	0.0000	0.0000	0.0002	0.3342
Lachnospiraceae UC5-1-2E3	0.0000	0.0000	0.0003	0.0002	0.0000	0.0006	**0.0380**
Olsenella	0.0003	0.0000	0.0007	0.0000	0.0000	0.0001	**0.0298**

Mycoplasma	0.0000	0.0000	0.0027	0.0004	0.0000	0.0027	**0.0249**
Lachnospiraceae AC2044 group	0.0000	0.0000	0.0013	0.0000	0.0000	0.0000	0.1709
Coriobacteriaceae UCG-002	0.0001	0.0000	0.0008	0.0000	0.0000	0.0002	0.0847
				**Week 4**			
		**DIET1**			**DIET2**		
	**Median**	**Min**	**Max**	**Median**	**Min**	**Max**	**p-value**
Alloprevotella	0.1146	0.0341	0.2013	0.0735	0.0258	0.1415	0.1036
Lachnoanaerobaculum	0.0194	0.0080	0.0347	0.0311	0.0161	0.0393	0.1893
Helicobacter	0.0410	0.0253	0.0704	0.0790	0.0246	0.1059	0.0313
Rikenellaceae RC9 gut group	0.0297	0.0090	0.0679	0.0302	0.0119	0.0442	0.7132
Candidatus Saccharimonas	0.0421	0.0263	0.0833	0.0597	0.0264	0.0858	0.3720
Bacteroides	0.1040	0.0654	0.1211	0.0865	0.0508	0.1449	0.3184
Prevotellaceae UCG-001	0.0910	0.0218	0.1158	0.0438	0.0140	0.0930	0.0406
Flavonifractor	0.0074	0.0038	0.0161	0.0169	0.0075	0.0342	0.0136
Colidextribacter	0.0363	0.0229	0.0790	0.0396	0.0303	0.0553	0.7132
Parasutterella	0.0479	0.0208	0.1005	0.0318	0.0222	0.0475	0.1563
Odoribacter	0.0139	0.0049	0.0308	0.0178	0.0090	0.0302	0.4948
Lachnospiraceae NK4A136 group	0.1649	0.0816	0.2491	0.1811	0.1119	0.1995	0.3720
Intestinimonas	0.0169	0.0068	0.0275	0.0167	0.0092	0.0234	0.9581
Mucispirillum	0.0062	0.0002	0.0144	0.0296	0.0107	0.0580	0.0028
Oscillospira	0.0028	0.0014	0.0089	0.0092	0.0051	0.0183	0.0074
Muribaculum	0.0476	0.0172	0.0951	0.0190	0.0117	0.0778	0.0181
Herbinix	0.0040	0.0022	0.0408	0.0062	0.0018	0.0217	0.7929
Negativibacillus	0.0033	0.0015	0.0073	0.0109	0.0064	0.0138	0.0019
Parabacteroides	0.0149	0.0063	0.0468	0.0195	0.0083	0.0365	0.7132
Alistipes	0.0092	0.0056	0.0216	0.0222	0.0070	0.0299	0.0313
Ruminococcus gnavus group	0.0061	0.0036	0.0154	0.0034	0.0020	0.0062	0.0181
Oscillibacter	0.0040	0.0014	0.0089	0.0104	0.0080	0.0233	0.0019
Ligilactobacillus	0.0068	0.0015	0.0158	0.0052	0.0022	0.0107	0.4309
Anaerotruncus	0.0004	0.0002	0.0011	0.0104	0.0061	0.0241	0.0009
Eubacterium xylanophilum group	0.0060	0.0022	0.0109	0.0049	0.0028	0.0090	0.8748
Prevotella 7	0.0010	0.0003	0.0033	0.0007	0.0002	0.0014	0.5635
Harryflintia	0.0011	0.0004	0.0025	0.0035	0.0019	0.0058	0.0039
Desulfovibrio	0.0145	0.0036	0.0211	0.0078	0.0046	0.0135	0.0313
Tuzzerella	0.0059	0.0038	0.0131	0.0082	0.0036	0.0142	0.2701
Anaeroplasma	0.0005	0.0004	0.0069	0.0008	0.0004	0.0038	0.7929
Butyricicoccaceae UCG-009	0.0029	0.0022	0.0047	0.0039	0.0017	0.0088	0.3720
Erysipelatoclostridium	0.0002	0.0000	0.0018	0.0002	0.0000	0.0010	0.9575
Ruminococcaceae UCG-001	0.0016	0.0000	0.0127	0.0011	0.0000	0.0119	1.0000
Lachnoclostridium	0.0059	0.0016	0.0117	0.0039	0.0023	0.0068	0.4948
Ruminococcaceae Incertae Sedis	0.0038	0.0023	0.0083	0.0063	0.0041	0.0081	0.1036
Butyricicoccus	0.0037	0.0029	0.0074	0.0020	0.0011	0.0052	**0.0181**
Stomatobaculum	0.0014	0.0000	0.0053	0.0019	0.0009	0.0080	0.4309
Prevotellaceae NK3B31 group	0.0000	0.0000	0.0000	0.0003	0.0000	0.0188	**0.0015**
Lactobacillus	0.0006	0.0002	0.0033	0.0017	0.0002	0.0197	0.2271

Eubacterium eligens group	0.0003	0.0000	0.0028	0.0019	0.0007	0.0067	**0.0180**
Lachnospiraceae GCA-900066575	0.0069	0.0036	0.0114	0.0025	0.0005	0.0036	**0.0014**
Rikenella	0.0024	0.0007	0.0034	0.0027	0.0018	0.0051	0.2271
Ruminococcus	0.0017	0.0009	0.0059	0.0053	0.0020	0.0061	**0.0313**
Acetatifactor	0.0054	0.0006	0.0148	0.0052	0.0013	0.0353	0.9581
Lactobacillaceae HT002	0.0003	0.0000	0.0013	0.0008	0.0000	0.0038	0.2630
Angelakisella	0.0007	0.0002	0.0038	0.0013	0.0004	0.0022	0.3720
Lachnospiraceae ASF356	0.0009	0.0002	0.0192	0.0060	0.0031	0.0183	**0.0181**
Lachnospiraceae UCG-006	0.0041	0.0024	0.0141	0.0016	0.0011	0.0045	**0.0313**
Oscillospiraceae UCG-003	0.0008	0.0002	0.0019	0.0006	0.0003	0.0019	0.5635
Eubacterium siraeum group	0.0009	0.0000	0.0041	0.0083	0.0042	0.0098	**0.0009**
Ruminococcaceae UBA1819	0.0000	0.0000	0.0000	0.0007	0.0000	0.0049	**0.0015**
Dubosiella	0.0031	0.0006	0.0055	0.0008	0.0001	0.0070	0.1278
Paludicola	0.0014	0.0002	0.0046	0.0027	0.0017	0.0074	**0.0313**
Lachnospiraceae FCS020 group	0.0003	0.0002	0.0019	0.0002	0.0000	0.0005	0.0822
Anaerosporobacter	0.0003	0.0000	0.0013	0.0002	0.0000	0.0013	0.8739
Bilophila	0.0036	0.0006	0.0066	0.0013	0.0007	0.0022	0.0239
Lachnospiraceae UCG-001	0.0017	0.0005	0.0059	0.0015	0.0004	0.0076	0.7132
Escherichia-Shigella	0.0019	0.0011	0.0046	0.0031	0.0021	0.0048	0.1893
Oscillospiraceae UCG-002	0.0002	0.0000	0.0007	0.0007	0.0003	0.0013	**0.0074**
Shuttleworthia	0.0000	0.0000	0.0002	0.0012	0.0006	0.0029	**0.0007**
Eubacterium nodatum group	0.0017	0.0012	0.0023	0.0045	0.0019	0.0064	**0.0019**
Oscillospiraceae V9D2013 group	0.0001	0.0000	0.0004	0.0010	0.0002	0.0016	**0.0036**
Oscillospiraceae NK4A214 group	0.0008	0.0000	0.0014	0.0007	0.0005	0.0012	0.5635
Anaerovorax	0.0009	0.0002	0.0017	0.0000	0.0000	0.0012	**0.0113**
Roseburia	0.0015	0.0002	0.0042	0.0050	0.0019	0.0106	**0.0136**
Lachnospiraceae UCG-003	0.0000	0.0000	0.0014	0.0002	0.0000	0.0008	0.2497
Ureaplasma	0.0000	0.0000	0.0000	0.0000	0.0000	0.0002	0.0764
Faecalibaculum	0.0004	0.0000	0.0066	0.0000	0.0000	0.0005	0.1448
Christensenellaceae R-7 group	0.0010	0.0002	0.0023	0.0009	0.0003	0.0019	0.9581
Anaerostipes	0.0004	0.0000	0.0008	0.0004	0.0001	0.0009	0.8748
Mobilitalea	0.0005	0.0000	0.0012	0.0000	0.0000	0.0007	**0.0136**
Lachnospiraceae A2	0.0017	0.0004	0.0062	0.0008	0.0002	0.0015	**0.0239**
Akkermansia	0.0000	0.0000	0.0049	0.0002	0.0000	0.0017	0.3318
Peptococcus	0.0011	0.0005	0.0027	0.0029	0.0009	0.0046	**0.0181**
Eubacterium fissicatena group	0.0010	0.0002	0.0036	0.0000	0.0000	0.0002	**0.0008**
Tyzzerella	0.0000	0.0000	0.0002	0.0014	0.0002	0.0046	**0.0008**
Lachnospiraceae UCG-009	0.0000	0.0000	0.0003	0.0001	0.0000	0.0017	0.5069
Monoglobus	0.0002	0.0000	0.0015	0.0008	0.0002	0.0013	0.1025
Anaerovoracaceae Family XIII UCG-001	0.0007	0.0002	0.0015	0.0004	0.0002	0.0012	0.1893
Marvinbryantia	0.0003	0.0000	0.0028	0.0001	0.0000	0.0002	0.1957
Salmonella	0.0001	0.0000	0.0004	0.0000	0.0000	0.0007	0.8622
Lactococcus	0.0000	0.0000	0.0009	0.0000	0.0000	0.0000	0.0764
Anaerovoracaceae Family XIII AD3011 group	0.0011	0.0005	0.0028	0.0006	0.0000	0.0016	**0.0406**
Lachnospiraceae UCG-004	0.0004	0.0000	0.0005	0.0002	0.0000	0.0009	0.4240
Veillonella	0.0004	0.0002	0.0013	0.0000	0.0000	0.0000	**0.0004**

Merdibacter	0.0000	0.0000	0.0003	0.0001	0.0000	0.0004	0.4328
Lachnospiraceae UC5-1-2E3	0.0002	0.0000	0.0006	0.0002	0.0000	0.0004	0.5968
Olsenella	0.0003	0.0000	0.0017	0.0000	0.0000	0.0005	0.1389
Mycoplasma	0.0000	0.0000	0.0000	0.0000	0.0000	0.0003	0.0764
Lachnospiraceae AC2044 group	0.0000	0.0000	0.0000	0.0001	0.0000	0.0004	**0.0325**
Coriobacteriaceae UCG-002	0.0000	0.0000	0.0010	0.0000	0.0000	0.0003	0.5656

	Week 12						
	**DIET1**			**DIET2**			
	**Median**	**Min**	**Max**	**Median**	**Min**	**Max**	**p-value**
Alloprevotella	0.0677	0.0351	0.2073	0.0382	0.0253	0.0781	0.0661
Lachnoanaerobaculum	0.0174	0.0044	0.0261	0.0224	0.0078	0.0471	0.1563
Helicobacter	0.0377	0.0185	0.0488	0.0532	0.0155	0.0774	0.1563
Rikenellaceae RC9 gut group	0.0218	0.0095	0.0346	0.0288	0.0098	0.0466	0.1036
Candidatus Saccharimonas	0.0472	0.0288	0.0888	0.0335	0.0024	0.0627	0.052
Bacteroides	0.0739	0.0337	0.2251	0.0772	0.0505	0.2352	0.7132
Prevotellaceae UCG-001	0.0467	0.0213	0.2070	0.0541	0.0254	0.1131	0.9581
Flavonifractor	0.0176	0.0092	0.0260	0.0223	0.0142	0.0265	0.1893
Colidextribacter	0.0402	0.0323	0.0503	0.0485	0.0361	0.0650	0.0661
Parasutterella	0.1050	0.0196	0.1580	0.0380	0.0086	0.0571	**0.0239**
Odoribacter	0.0173	0.0052	0.0624	0.0232	0.0132	0.0380	0.1278
Lachnospiraceae NK4A136 group	0.1571	0.1080	0.1956	0.2291	0.1457	0.3176	**0.0074**
Intestinimonas	0.0190	0.0110	0.0417	0.0197	0.0111	0.0297	0.7929
Mucispirillum	0.0052	0.0018	0.0118	0.0208	0.0112	0.0321	**0.0014**
Oscillospira	0.0054	0.0025	0.0102	0.0076	0.0008	0.0147	0.0831
Muribaculum	0.0467	0.0210	0.0843	0.0232	0.0043	0.0441	**0.0181**
Herbinix	0.0065	0.0019	0.0275	0.0067	0.0012	0.0170	0.7929
Negativibacillus	0.0036	0.0009	0.0106	0.0108	0.0016	0.0161	**0.0239**
Parabacteroides	0.0122	0.0026	0.0467	0.0102	0.0053	0.0393	0.4309
Alistipes	0.0142	0.0068	0.0459	0.0149	0.0063	0.0574	0.6365
Ruminococcus gnavus group	0.0021	0.0009	0.0050	0.0024	0.0009	0.0040	1.0000
Oscillibacter	0.0071	0.0021	0.0106	0.0124	0.0040	0.0170	**0.0181**
Ligilactobacillus	0.0204	0.0025	0.0358	0.0028	0.0004	0.0041	**0.0039**
Anaerotruncus	0.0000	0.0000	0.0007	0.0071	0.0008	0.0228	**0.0008**
Eubacterium xylanophilum group	0.0082	0.0042	0.0196	0.0063	0.0021	0.0133	0.2271
Prevotella 7	0.0008	0.0000	0.0047	0.0011	0.0000	0.0029	0.5624
Harryflintia	0.0015	0.0005	0.0040	0.0037	0.0012	0.0057	**0.0136**
Desulfovibrio	0.0088	0.0048	0.0351	0.0136	0.0061	0.0285	0.372
Tuzzerella	0.0044	0.0030	0.0091	0.0142	0.0065	0.0210	**0.0054**
Anaeroplasma	0.0028	0.0013	0.0046	0.0016	0.0008	0.0026	0.0831
Butyricicoccaceae UCG-009	0.0028	0.0013	0.0062	0.0021	0.0011	0.0132	0.7929
Erysipelatoclostridium	0.0000	0.0000	0.0011	0.0000	0.0000	0.0002	0.4066
Ruminococcaceae UCG-001	0.0002	0.0000	0.0046	0.0003	0.0000	0.0037	0.9575
Lachnoclostridium	0.0050	0.0023	0.0083	0.0055	0.0012	0.0129	0.8748
Ruminococcaceae Incertae Sedis	0.0024	0.0013	0.0064	0.0032	0.0018	0.0048	0.4309
Butyricicoccus	0.0032	0.0019	0.0095	0.0021	0.0012	0.0037	**0.0313**

Stomatobaculum	0.0024	0.0000	0.0075	0.0033	0.0006	0.0051	0.7132
Prevotellaceae NK3B31 group	0.0000	0.0000	0.0040	0.0000	0.0000	0.0009	0.7001
Lactobacillus	0.0025	0.0003	0.0051	0.0014	0.0000	0.0028	0.2271
Eubacterium eligens group	0.0004	0.0000	0.0060	0.0004	0.0000	0.0021	1.0000
Lachnospiraceae GCA-900066575	0.0059	0.0026	0.0185	0.0028	0.0022	0.0077	0.1278
Rikenella	0.0020	0.0003	0.0043	0.0040	0.0014	0.0083	**0.0181**
Ruminococcus	0.0001	0.0000	0.0094	0.0004	0.0000	0.0009	0.7059
Acetatifactor	0.0083	0.0034	0.0121	0.0061	0.0037	0.0366	0.6365
Lactobacillaceae HT002	0.0013	0.0002	0.0029	0.0004	0.0000	0.0019	0.1893
Angelakisella	0.0011	0.0002	0.0020	0.0007	0.0002	0.0024	0.4309
Lachnospiraceae ASF356	0.0012	0.0000	0.0025	0.0066	0.0001	0.0193	**0.0313**
Lachnospiraceae UCG-006	0.0041	0.0013	0.0084	0.0018	0.0007	0.0022	0.0661
Oscillospiraceae UCG-003	0.0016	0.0008	0.0054	0.0010	0.0006	0.0016	**0.0406**
Eubacterium siraeum group	0.0001	0.0000	0.0019	0.0000	0.0000	0.0003	0.1105
Ruminococcaceae UBA1819	0.0000	0.0000	0.0000	0.0002	0.0000	0.0021	**0.0046**
Dubosiella	0.0030	0.0000	0.0095	0.0002	0.0000	0.0026	**0.0122**
Paludicola	0.0015	0.0006	0.0037	0.0013	0.0002	0.0017	0.4309
Lachnospiraceae FCS020 group	0.0009	0.0003	0.0022	0.0003	0.0000	0.0009	0.0181
Anaerosporobacter	0.0015	0.0002	0.0031	0.0002	0.0000	0.0005	**0.0100**
Bilophila	0.0012	0.0000	0.0044	0.0034	0.0012	0.0116	0.1036
Lachnospiraceae UCG-001	0.0010	0.0000	0.0113	0.0009	0.0003	0.0021	0.7929
Escherichia-Shigella	0.0001	0.0000	0.0005	0.0003	0.0000	0.0076	0.1222
Oscillospiraceae UCG-002	0.0004	0.0002	0.0018	0.0010	0.0003	0.0038	0.1563
Shuttleworthia	0.0011	0.0000	0.0092	0.0010	0.0001	0.0047	0.9581
Eubacterium nodatum group	0.0026	0.0015	0.0057	0.0028	0.0005	0.0062	1.0000
Oscillospiraceae V9D2013 group	0.0005	0.0000	0.0011	0.0006	0.0002	0.0012	0.8748
Oscillospiraceae NK4A214 group	0.0007	0.0003	0.0022	0.0008	0.0003	0.0018	0.9581
Anaerovorax	0.0006	0.0000	0.0015	0.0019	0.0008	0.0045	**0.0101**
Roseburia	0.0031	0.0014	0.0042	0.0038	0.0014	0.0086	0.3184
Lachnospiraceae UCG-003	0.0000	0.0000	0.0000	0.0008	0.0000	0.0050	**0.0046**
Ureaplasma	0.0002	0.0000	0.0008	0.0000	0.0000	0.0000	**0.0128**
Faecalibaculum	0.0017	0.0002	0.0175	0.0000	0.0000	0.0000	**0.0004**
Christensenellaceae R-7 group	0.0012	0.0006	0.0029	0.0004	0.0000	0.0032	**0.0239**
Anaerostipes	0.0003	0.0000	0.0008	0.0001	0.0000	0.0008	0.1827
Mobilitalea	0.0003	0.0001	0.0022	0.0001	0.0000	0.0008	0.1250
Lachnospiraceae A2	0.0014	0.0008	0.0020	0.0016	0.0002	0.0044	0.7132
Akkermansia	0.0005	0.0000	0.0030	0.0000	0.0000	0.0005	0.0728
Peptococcus	0.0024	0.0008	0.0036	0.0030	0.0023	0.0057	0.0831
Eubacterium fissicatena group	0.0007	0.0002	0.0019	0.0000	0.0000	0.0003	**0.0034**
Tyzzerella	0.0000	0.0000	0.0006	0.0013	0.0003	0.0048	**0.0015**
Lachnospiraceae UCG-009	0.0005	0.0000	0.0018	0.0001	0.0000	0.0002	**0.0111**
Monoglobus	0.0009	0.0000	0.0031	0.0002	0.0000	0.0012	0.155
Anaerovoracaceae Family XIII UCG-001	0.0008	0.0002	0.0010	0.0005	0.0000	0.0014	0.4948
Marvinbryantia	0.0000	0.0000	0.0005	0.0000	0.0000	0.0008	0.8360
Salmonella	0.0001	0.0000	0.0006	0.0002	0.0000	0.0038	0.7840
Lactococcus	0.0000	0.0000	0.0005	0.0000	0.0000	0.0000	0.0764

Anaerovoracaceae Family XIII AD3011 group	0.0011	0.0006	0.0022	0.0004	0.0001	0.0016	0.052
Lachnospiraceae UCG-004	0.0004	0.0002	0.0007	0.0003	0.0000	0.0017	0.9581
Veillonella	0.0007	0.0000	0.0015	0.0000	0.0000	0.0000	**0.0015**
Merdibacter	0.0000	0.0000	0.0000	0.0000	0.0000	0.0002	0.3816
Lachnospiraceae UC5-1-2E3	0.0002	0.0000	0.0006	0.0001	0.0000	0.0004	0.2630
Olsenella	0.0002	0.0000	0.0015	0.0000	0.0000	0.0000	**0.0046**
Mycoplasma	0.0000	0.0000	0.0003	0.0000	0.0000	0.0000	0.3816
Lachnospiraceae AC2044 group	0.0000	0.0000	0.0000	0.0001	0.0000	0.0011	**0.0128**
Coriobacteriaceae UCG-002	0.0000	0.0000	0.0005	0.0000	0.0000	0.0002	0.2143
				**Week 24**			
		**DIET1**			**DIET2**		
	**Median**	**Min**	**Max**	**Median**	**Min**	**Max**	**p-value**
Alloprevotella	0.0792	0.0043	0.1869	0.0584	0.0248	0.1891	0.8748
Lachnoanaerobaculum	0.0085	0.0022	0.0237	0.0265	0.0103	0.1028	**0.0074**
Helicobacter	0.0451	0.0119	0.0702	0.0283	0.0065	0.0655	0.1036
Rikenellaceae RC9 gut group	0.0376	0.0138	0.1366	0.0171	0.0053	0.0288	0.0661
Candidatus Saccharimonas	0.0438	0.0121	0.0573	0.0617	0.0366	0.2069	**0.0181**
Bacteroides	0.1807	0.0824	0.4089	0.1238	0.0299	0.1990	0.1278
Prevotellaceae UCG-001	0.0548	0.0108	0.1315	0.0352	0.0000	0.0733	0.2701
Flavonifractor	0.0145	0.0071	0.0181	0.0167	0.0112	0.0214	0.2271
Colidextribacter	0.0340	0.0192	0.0519	0.0396	0.0248	0.0511	0.5635
Parasutterella	0.0396	0.0047	0.0820	0.0504	0.0368	0.0716	0.2271
Odoribacter	0.0180	0.0065	0.0298	0.0091	0.0054	0.0196	0.0831
Lachnospiraceae NK4A136 group	0.1037	0.0276	0.2714	0.1476	0.0969	0.1740	0.2271
Intestinimonas	0.0151	0.0091	0.0501	0.0223	0.0098	0.0329	0.7929
Mucispirillum	0.0057	0.0010	0.0128	0.0116	0.0004	0.0227	0.3720
Oscillospira	0.0045	0.0028	0.0155	0.0053	0.0034	0.0108	0.6365
Muribaculum	0.0319	0.0178	0.1109	0.0331	0.0146	0.0504	0.9581
Herbinix	0.0018	0.0004	0.0222	0.0057	0.0013	0.0090	0.2271
Negativibacillus	0.0013	0.0000	0.0056	0.0087	0.0054	0.0144	**0.0014**
Parabacteroides	0.0301	0.0132	0.1131	0.0172	0.0028	0.0436	0.1036
Alistipes	0.0223	0.0125	0.0381	0.0102	0.0034	0.0232	**0.0074**
Ruminococcus gnavus group	0.0029	0.0012	0.0086	0.0043	0.0021	0.0148	0.2701
Oscillibacter	0.0080	0.0054	0.0147	0.0103	0.0080	0.0153	0.1893
Ligilactobacillus	0.0033	0.0007	0.0129	0.0142	0.0054	0.1214	**0.0074**
Anaerotruncus	0.0001	0.0000	0.0003	0.0052	0.0010	0.0155	**0.0009**
Eubacterium xylanophilum group	0.0061	0.0008	0.0109	0.0067	0.0021	0.0168	0.3184
Prevotella 7	0.0016	0.0005	0.0032	0.0003	0.0000	0.0019	**0.0308**
Harryflintia	0.0015	0.0004	0.0029	0.0047	0.0015	0.0087	**0.0039**
Desulfovibrio	0.0098	0.0013	0.0233	0.0115	0.0071	0.0323	0.2271
Tuzzerella	0.0061	0.0022	0.0106	0.0058	0.0034	0.0179	0.4948
Anaeroplasma	0.0026	0.0013	0.0128	0.0005	0.0001	0.0022	**0.0028**
Butyricicoccaceae UCG-009	0.0019	0.0004	0.0028	0.0041	0.0012	0.0117	**0.0136**
Erysipelatoclostridium	0.0014	0.0000	0.0066	0.0000	0.0000	0.0019	0.1783
Ruminococcaceae UCG-001	0.0000	0.0000	0.0003	0.0025	0.0000	0.0193	0.0924

Lachnoclostridium	0.0078	0.0021	0.0224	0.0088	0.0027	0.0144	1.0000
Ruminococcaceae Incertae Sedis	0.0028	0.0019	0.0070	0.0073	0.0028	0.0100	**0.0239**
Butyricicoccus	0.0040	0.0015	0.0156	0.0027	0.0006	0.0063	0.3184
Stomatobaculum	0.0015	0.0000	0.0102	0.0008	0.0000	0.0142	0.4619
Prevotellaceae NK3B31 group	0.0007	0.0000	0.0406	0.0000	0.0000	0.0020	0.0550
Lactobacillus	0.0002	0.0000	0.0014	0.0147	0.0023	0.0438	**0.0009**
Eubacterium eligens group	0.0001	0.0000	0.0057	0.0002	0.0000	0.0025	0.7059
Lachnospiraceae GCA-900066575	0.0065	0.0035	0.0148	0.0041	0.0029	0.0067	0.0661
Rikenella	0.0013	0.0000	0.0061	0.0028	0.0011	0.0106	0.1278
Ruminococcus	0.0016	0.0000	0.0050	0.0024	0.0005	0.0065	0.2701
Acetatifactor	0.0036	0.0002	0.0077	0.0044	0.0021	0.0070	0.6365
Lactobacillaceae HT002	0.0001	0.0000	0.0005	0.0059	0.0013	0.0136	**0.0009**
Angelakisella	0.0010	0.0002	0.0055	0.0007	0.0002	0.0018	0.4948
Lachnospiraceae ASF356	0.0001	0.0000	0.0119	0.0223	0.0075	0.0337	**0.0018**
Lachnospiraceae UCG-006	0.0034	0.0009	0.0110	0.0021	0.0012	0.0061	0.3720
Oscillospiraceae UCG-003	0.0021	0.0003	0.0089	0.0009	0.0001	0.0029	0.1278
Eubacterium siraeum group	0.0011	0.0000	0.0037	0.0006	0.0000	0.0050	0.7923
Ruminococcaceae UBA1819	0.0000	0.0000	0.0000	0.0004	0.0000	0.0075	**0.0325**
Dubosiella	0.0028	0.0003	0.0795	0.0011	0.0000	0.0020	0.1893
Paludicola	0.0009	0.0000	0.0022	0.0014	0.0010	0.0029	0.1563
Lachnospiraceae FCS020 group	0.0007	0.0000	0.0035	0.0001	0.0000	0.0007	0.1706
Anaerosporobacter	0.0002	0.0000	0.0012	0.0001	0.0000	0.0005	0.3514
Bilophila	0.0035	0.0005	0.0070	0.0009	0.0006	0.0031	0.0661
Lachnospiraceae UCG-001	0.0006	0.0000	0.0017	0.0011	0.0000	0.0089	0.2687
Escherichia-Shigella	0.0006	0.0002	0.0022	0.0006	0.0000	0.0085	0.9581
Oscillospiraceae UCG-002	0.0003	0.0000	0.0024	0.0008	0.0002	0.0054	0.1278
Shuttleworthia	0.0000	0.0000	0.0003	0.0000	0.0000	0.0001	1.0000
Eubacterium nodatum group	0.0016	0.0006	0.0071	0.0083	0.0021	0.0123	**0.0054**
Oscillospiraceae V9D2013 group	0.0004	0.0000	0.0017	0.0002	0.0000	0.0014	0.5283
Oscillospiraceae NK4A214 group	0.0004	0.0002	0.0008	0.0011	0.0006	0.0017	**0.0014**
Anaerovorax	0.0006	0.0000	0.0012	0.0024	0.0010	0.0031	**0.0014**
Roseburia	0.0031	0.0006	0.0050	0.0044	0.0008	0.0069	0.1893
Lachnospiraceae UCG-003	0.0000	0.0000	0.0014	0.0003	0.0000	0.0119	0.0535
Ureaplasma	0.0001	0.0000	0.0003	0.0000	0.0000	0.0002	0.2716
Faecalibaculum	0.0000	0.0000	0.0017	0.0000	0.0000	0.0005	0.9491
Christensenellaceae R-7 group	0.0004	0.0000	0.0029	0.0020	0.0013	0.0070	**0.0136**
Anaerostipes	0.0001	0.0000	0.0003	0.0004	0.0000	0.0008	**0.0486**
Mobilitalea	0.0003	0.0000	0.0014	0.0001	0.0000	0.0003	0.1706
Lachnospiraceae A2	0.0010	0.0001	0.0033	0.0007	0.0004	0.0016	0.7929
Akkermansia	0.0009	0.0000	0.0071	0.0000	0.0000	0.0026	0.3253
Peptococcus	0.0014	0.0000	0.0026	0.0028	0.0015	0.0065	**0.0054**
Eubacterium fissicatena group	0.0006	0.0000	0.0026	0.0004	0.0002	0.0011	1.0000
Tyzzerella	0.0000	0.0000	0.0000	0.0001	0.0000	0.0014	**0.0325**
Lachnospiraceae UCG-009	0.0002	0.0000	0.0010	0.0000	0.0000	0.0006	0.3695
Monoglobus	0.0001	0.0000	0.0017	0.0012	0.0004	0.0019	**0.0178**
Anaerovoracaceae Family XIII UCG-001	0.0004	0.0001	0.0010	0.0014	0.0003	0.0020	**0.0054**

Marvinbryantia	0.0000	0.0000	0.0005	0.0000	0.0000	0.0002	0.4066
Salmonella	0.0002	0.0000	0.0004	0.0000	0.0000	0.0002	**0.0496**
Lactococcus	0.0000	0.0000	0.0002	0.0000	0.0000	0.0000	0.1709
Anaerovoracaceae Family XIII AD3011 group	0.0007	0.0002	0.0018	0.0011	0.0003	0.0026	0.2271
Lachnospiraceae UCG-004	0.0002	0.0000	0.0006	0.0007	0.0002	0.0010	**0.0101**
Veillonella	0.0001	0.0000	0.0011	0.0000	0.0000	0.0002	0.0535
Merdibacter	0.0000	0.0000	0.0002	0.0000	0.0000	0.0014	0.3342
Lachnospiraceae UC5-1-2E3	0.0000	0.0000	0.0005	0.0001	0.0000	0.0005	0.7723
Olsenella	0.0002	0.0000	0.0006	0.0000	0.0000	0.0007	0.2976
Mycoplasma	0.0000	0.0000	0.0000	0.0000	0.0000	0.0006	0.3816
Lachnospiraceae AC2044 group	0.0000	0.0000	0.0000	0.0003	0.0000	0.0009	**0.0046**
Coriobacteriaceae UCG-002	0.0002	0.0000	0.0005	0.0000	0.0000	0.0002	**0.0402**
Faecalibacterium	0.0003	0.0000	0.0006	0.0000	0.0000	0.0004	**0.0496**

Supplementary Table S3: Relative abundance comparison between diet groups at Genus level in the different weeks of study. Significant differences established using the Wilcoxon signed-rank test ($p<0.05$). p-values in bold style were statistically significant.

Supplementary Table S4: Blood metabolic parameters comparison

Supplementary Table S4: *Blood metabolic parameters comparison*

	**DIET 1**		**DIET 2**		
	**Mean**	**SD**	**Mean**	**SD**	**p-value**
Body weight (g)	34.6	3.0	49.6	5.0	**0.0039**
VAT weight (g)	1.3	0.2	2.5	0.3	**0.0007**
Glucose (mg/dL)	169	34	187	18	0.3882
Cholesterol (mg/dL)	128	13	214	11	**5.641e-05**
Triglycerides (mg/dL)	78	14	113	11	**0.0095**
HDL-c (mg/dL)	101	11	178	9	**4.011e-05**
LDL-c (mg/dL)	12	2	14	7	0.4952
AST (UI/L)	71	38	87	33	0.5497
ALT (UI/L)	15	7	19	13	0.6066
Leptin (pg/mL)	13286	9891	51042	1421	**0.0042**
Adiponectin (ng/mL)	3266	534	3209	180	0.8505

Supplementary Table S4: Body and VAT weight and Blood metabolic parameters comparison for groups D1 and D2 at week 24 of feeding. Significant differences established using two-tailed Student's t-test ($p<0.05$). p-values in bold style were statistically significant.

Abbreviations: SD: standard deviation, VAT: visceral adipose tissue, HDL-c: high-density lipoprotein cholesterol, LDL-c: low-density lipoprotein cholesterol, AST: aspartate aminotransferase, ALT: alanine aminotransferase

<u>Supplementary Table S5:</u> <u>Immune cells population in TAV comparison</u>

Supplementary Table S5: Immune cells population in TAV comparison

	DIET 1		DIET 2		
Parameter	**Mean**	**SD**	**Mean**	**SD**	**p-value**
% CD11b+	32.7	11.5	5.5	3.1	**0.0150**
% CD11b+ CD36+	48.9	16.8	20.0	6.8	**0.0336**
% CD11b+ F4/80+	31.3	12.0	10.9	5.7	**0.0342**
% CD11b+ F4/80+ CD36+	55.8	9.3	32.9	6.0	**0.0086**
% CD11b+ F4/80+ CD206- CD11c+	40.0	9.7	21.7	5.0	**0.0233**
MFI CD11b+ F4/80+ CD206- CD11c+ ROS	46.8	14.9	16.4	4.6	**0.0221**
% CD11b+ F4/80+ CD206+ CD11c+	24.1	8.1	13.6	1.8	**0.0221**
% CD11b+ F4/80+ CD2066 CD11c-	0.3	0.2	0.3	0.2	1.0000
% CD11b+ F4/80+ CD206- CD11c-	12.7	7.8	5.7	2.8	0.1717
MFI CD11b+ F4/80+ ROS	46.4	15.7	15.5	3.9	**0.0258**
MFI CD11b+ ROS	37.0	14.8	8.2	2.6	**0.0281**
% CD11b-	41.2	11.5	35.1	5.3	0.3869
% CD11b- CD36+	35.6	20.7	3.9	2.2	0.0540
% Leukocytes CD36+	42.4	18.9	6.4	4.1	**0.0288**
MFI Leukocytes ROS	34.4	11.9	3.5	2.1	**0.0126**

Supplementary Table S5: Comparison immune cells population in VAT for D1 and D2 groups at week 24 of feeding. Significant differences established using two-tailed Student's t test ($p<0.05$). p-values in bold style were statistically significant.

<u>Abbreviations:</u> SD: standard deviation range, CD: cluster of differentiation, ROS: reactive oxygen species.

Supplementary Table S6: variables showing significant correlation

Supplementary Table S6: *variables showing significant correlation*

Genus	Variable	r	p-value
Candidatus Saccharimonas	Body Weight	0,74	0,0365
Negativibacillus	Body Weight	0,77	0,0245
Anaerotruncus	Body Weight	0,9	0,0023
Harryflintia	Body Weight	0,72	0,0445
Butyricicoccaceae UCG-009	Body Weight	0,72	0,0431
Ruminococcaceae Incertae Sedis	Body Weight	0,71	0,0476
Alistipes	Body Weight	-0,73	0,0414
Lactobacillus	Body Weight	0,85	0,0074
Lachnospiraceae GCA-900066575	Body Weight	-0,78	0,021
Lactobacillaceae HT002	Body Weight	0,82	0,0133
Lachnospiraceae ASF356	Body Weight	0,85	0,0081
Anaerovorax	Body Weight	0,92	0,0012
Peptococcus	Body Weight	0,77	0,0252
Anaerostipes	Body Weight	0,78	0,0233
Monoglobus	Body Weight	0,77	0,0268
Lachnospiraceae UCG-004	Body Weight	0,77	0,0248
Lachnospiraceae AC2044 group	Body Weight	0,79	0,0199
Negativibacillus	VAT Weight	0,81	0,014
Anaerotruncus	VAT Weight	0,87	0,0054
Harryflintia	VAT Weight	0,71	0,0479
Ruminococcaceae Incertae Sedis	VAT Weight	0,73	0,0394
Alistipes	VAT Weight	-0,73	0,0416
Lactobacillus	VAT Weight	0,89	0,0033
Lachnospiraceae GCA-900066575	VAT Weight	-0,79	0,0207
Lactobacillaceae HT002	VAT Weight	0,84	0,0087
Lachnospiraceae ASF356	VAT Weight	0,85	0,0071
Anaerovorax	VAT Weight	0,88	0,0037
Peptococcus	VAT Weight	0,76	0,0274
Anaerostipes	VAT Weight	0,8	0,0171
Monoglobus	VAT Weight	0,75	0,0324
Lachnospiraceae UCG-004	VAT Weight	0,78	0,0237
Lachnospiraceae AC2044 group	VAT Weight	0,73	0,0418
Muribaculum	Glucose	0,71	0,0485
Odoribacter	Glucose	-0,79	0,0195
Anaeroplasma	Glucose	-0,85	0,0075
Prevotellaceae NK3B31 group	Glucose	-0,75	0,0315
Eubacterium eligens group	Glucose	-0,8	0,0179
Angelakisella	Glucose	-0,83	0,011
Lachnospiraceae FCS020 group	Glucose	-0,82	0,0127
Bilophila	Glucose	-0,87	0,0052
Negativibacillus	Cholesterol	0,81	0,0159
Ligilactobacillus	Cholesterol	0,87	0,0045
Ruminococcaceae Incertae Sedis	Cholesterol	0,78	0,0232

Lactobacillus	Cholesterol	0,89	0,0034
Lachnospiraceae GCA-900066575	Cholesterol	-0,73	0,0387
Lactobacillaceae HT002	Cholesterol	0,8	0,0177
Lachnospiraceae ASF356	Cholesterol	0,76	0,0302
Peptococcus	Cholesterol	0,75	0,033
Christensenellaceae R-7 group	Cholesterol	0,74	0,0347
Anaerostipes	Cholesterol	0,86	0,0061
Lachnospiraceae UCG-004	Cholesterol	0,72	0,0438
Lachnoanaerobaculum	Triglycerides	0,74	0,0361
Rikenellaceae RC9 gut group	Triglycerides	-0,74	0,0368
Negativibacillus	Triglycerides	0,98	1.25E-05
Ligilactobacillus	Triglycerides	0,71	0,0479
Harryflintia	Triglycerides	0,82	0,0126
Lactobacillus	Triglycerides	0,88	0,0044
Lactobacillaceae HT002	Triglycerides	0,86	0,0065
Oscillospiraceae UCG-003	Triglycerides	-0,75	0,0308
Oscillospiraceae NK4A214 group	Triglycerides	0,72	0,0459
Peptococcus	Triglycerides	0,72	0,0444
Christensenellaceae R-7 group	Triglycerides	0,8	0,0181
Lachnospiraceae UCG-004	Triglycerides	0,88	0,0044
Negativibacillus	HDL-c	0,8	0,0172
Ligilactobacillus	HDL-c	0,9	0,0021
Ruminococcaceae Incertae Sedis	HDL-c	0,74	0,0371
Alistipes	HDL-c	-0,72	0,0455
Lactobacillus	HDL-c	0,88	0,0038
Lachnospiraceae GCA-900066575	HDL-c	-0,72	0,0432
Lactobacillaceae HT002	HDL-c	0,8	0,0171
Eubacterium nodatum group	HDL-c	0,72	0,0432
Peptococcus	HDL-c	0,78	0,0233
Christensenellaceae R-7 group	HDL-c	0,77	0,0269
Anaerostipes	HDL-c	0,85	0,008
Lachnospiraceae UCG-004	HDL-c	0,72	0,0419
Ruminococcaceae Incertae Sedis	LDL-c	0,75	0,033
Alloprevotella	AST	0,86	0,0063
Odoribacter	AST	-0,73	0,0382
Escherichia-Shigella	AST	0,81	0,0147
Oscillospiraceae V9D2013 group	AST	-0,81	0,0139
Escherichia-Shigella	ALT	0,83	0,01
Christensenellaceae R-7 group	ALT	0,79	0,0191
Rikenellaceae RC9 gut group	Leptin	-0,72	0,0444
Negativibacillus	Leptin	0,83	0,0099
Ligilactobacillus	Leptin	0,82	0,0127
Anaerotruncus	Leptin	0,72	0,0454
Ruminococcaceae Incertae Sedis	Leptin	0,73	0,0395
Alistipes	Leptin	-0,77	0,0255
Lactobacillus	Leptin	0,89	0,0028

Lachnospiraceae GCA-900066575	Leptin	-0,76	0,0276
Lactobacillaceae HT002	Leptin	0,83	0,0107
Eubacterium nodatum group	Leptin	0,72	0,0425
Lachnospiraceae ASF356	Leptin	0,78	0,0235
Anaerovorax	Leptin	0,8	0,0183
Peptococcus	Leptin	0,85	0,0069
Christensenellaceae R-7 group	Leptin	0,76	0,03
Anaerostipes	Leptin	0,79	0,019
Lachnospiraceae UCG-004	Leptin	0,83	0,0105
Eubacterium xylanophilum group	Adiponectin	0,71	0,048
Lachnospiraceae FCS020 group	Adiponectin	0,82	0,0125
Eubacterium fissicatena group	Adiponectin	0,78	0,0211
Salmonella	Adiponectin	0,81	0,0157

**Genus**	**Inmune cells variable**	**r**	**p-value**
Lachnoanaerobaculum	% CD11b+	-0,76	0,0291
Rikenellaceae RC9 gut group	% CD11b+	0,88	0,0038
Negativibacillus	% CD11b+	-0,8	0,0162
Ligilactobacillus	% CD11b+	-0,74	0,0346
Parabacteroides	% CD11b+	0,79	0,0191
Alistipes	% CD11b+	0,78	0,0216
Lactobacillus	% CD11b+	-0,8	0,0179
Lactobacillaceae HT002	% CD11b+	-0,75	0,0337
Eubacterium nodatum group	% CD11b+	-0,77	0,0248
Anaerovorax	% CD11b+	-0,77	0,0241
Peptococcus	% CD11b+	-0,84	0,0087
Christensenellaceae R-7 group	% CD11b+	-0,73	0,0391
Anaerostipes	% CD11b+	-0,73	0,0383
Lachnospiraceae UCG-004	% CD11b+	-0,83	0,0106
Anaerovoracaceae Family XIII UCG-001	% CD11b+	-0,79	0,0202
Lachnoanaerobaculum	% CD11b+ CD36+	-0,74	0,0359
Rikenellaceae RC9 gut group	% CD11b+ CD36+	0,91	0,0019
Bacteroides	% CD11b+ CD36+	0,84	0,0096
Parabacteroides	% CD11b+ CD36+	0,89	0,0028
Desulfovibrio	% CD11b+ CD36+	-0,71	0,0484
Butyricicoccaceae UCG-009	% CD11b+ CD36+	-0,74	0,0371
Tuzzerella	% CD11b+ CD36+	-0,75	0,0303
Eubacterium nodatum group	% CD11b+ CD36+	-0,72	0,0452
Anaerovorax	% CD11b+ CD36+	-0,78	0,0222
Peptococcus	% CD11b+ CD36+	-0,74	0,0369
Monoglobus	% CD11b+ CD36+	-0,74	0,0342
Lachnospiraceae UCG-004	% CD11b+ CD36+	-0,74	0,0364
Anaerovoracaceae Family XIII UCG-001	% CD11b+ CD36+	-0,8	0,0162
Lachnoanaerobaculum	% CD11b+ F4/80+	-0,76	0,0271
Rikenellaceae RC9 gut group	% CD11b+ F4/80+	0,92	0,001
Bacteroides	% CD11b+ F4/80+	0,77	0,0255

Negativibacillus	% CD11b+ F4/80+	-0,74	0,0362
Parabacteroides	% CD11b+ F4/80+	0,85	0,0072
Alistipes	% CD11b+ F4/80+	0,87	0,005
Eubacterium nodatum group	% CD11b+ F4/80+	-0,82	0,0125
Anaerovorax	% CD11b+ F4/80+	-0,75	0,0311
Peptococcus	% CD11b+ F4/80+	-0,83	0,0101
Anaerostipes	% CD11b+ F4/80+	-0,72	0,0462
Lachnospiraceae UCG-004	% CD11b+ F4/80+	-0,82	0,0135
Anaerovoracaceae Family XIII UCG-001	% CD11b+ F4/80+	-0,85	0,0069
Rikenellaceae RC9 gut group	% CD11b+ F4/80+ CD36+	0,86	0,0067
Bacteroides	% CD11b+ F4/80+ CD36+	0,71	0,0477
Negativibacillus	% CD11b+ F4/80+ CD36+	-0,76	0,0279
Oscillibacter	% CD11b+ F4/80+ CD36+	-0,72	0,0446
Anaerotruncus	% CD11b+ F4/80+ CD36+	-0,79	0,02
Parabacteroides	% CD11b+ F4/80+ CD36+	0,78	0,0235
Harryflintia	% CD11b+ F4/80+ CD36+	-0,74	0,0378
Butyricicoccaceae UCG-009	% CD11b+ F4/80+ CD36+	-0,75	0,0329
Ruminococcaceae Incertae Sedis	% CD11b+ F4/80+ CD36+	-0,79	0,0209
Alistipes	% CD11b+ F4/80+ CD36+	0,71	0,048
Lactobacillus	% CD11b+ F4/80+ CD36+	-0,79	0,0199
Tuzzerella	% CD11b+ F4/80+ CD36+	-0,71	0,0481
Lactobacillaceae HT002	% CD11b+ F4/80+ CD36+	-0,74	0,0352
Lachnospiraceae ASF356	% CD11b+ F4/80+ CD36+	-0,78	0,0231
Anaerovorax	% CD11b+ F4/80+ CD36+	-0,88	0,0039
Peptococcus	% CD11b+ F4/80+ CD36+	-0,73	0,0408
Anaerostipes	% CD11b+ F4/80+ CD36+	-0,77	0,0252
Monoglobus	% CD11b+ F4/80+ CD36+	-0,76	0,0292
Lachnospiraceae UCG-004	% CD11b+ F4/80+ CD36+	-0,77	0,0262
Anaerovoracaceae Family XIII UCG-001	% CD11b+ F4/80+ CD36+	-0,72	0,0452
Rikenellaceae RC9 gut group	% CD11b+ F4/80+ CD206- CD11c+	0,72	0,0433
Negativibacillus	% CD11b+ F4/80+ CD206- CD11c+	-0,79	0,0202
Anaerotruncus	% CD11b+ F4/80+ CD206- CD11c+	-0,71	0,0495
Alistipes	% CD11b+ F4/80+ CD206- CD11c+	0,9	0,0025
Lactobacillus	% CD11b+ F4/80+ CD206- CD11c+	-0,71	0,0494
Lachnospiraceae GCA-900066575	% CD11b+ F4/80+ CD206- CD11c+	0,81	0,0156
Eubacterium nodatum group	% CD11b+ F4/80+ CD206- CD11c+	-0,72	0,0446
Lachnospiraceae ASF356	% CD11b+ F4/80+ CD206- CD11c+	-0,84	0,0097
Anaerovorax	% CD11b+ F4/80+ CD206- CD11c+	-0,78	0,0222
Peptococcus	% CD11b+ F4/80+ CD206- CD11c+	-0,78	0,0231
Anaerostipes	% CD11b+ F4/80+ CD206- CD11c+	-0,76	0,0279
Lachnospiraceae UCG-004	% CD11b+ F4/80+ CD206- CD11c+	-0,8	0,016
Lachnoanaerobaculum	MFI CD11b+ F4/80+ CD206- CD11c+ ROS	-0,71	0,0492
Negativibacillus	MFI CD11b+ F4/80+ CD206- CD11c+ ROS	-0,83	0,0114
Ligilactobacillus	MFI CD11b+ F4/80+ CD206- CD11c+ ROS	-0,79	0,021
Anaeroplasma	MFI CD11b+ F4/80+ CD206- CD11c+ ROS	0,77	0,0245
Alistipes	MFI CD11b+ F4/80+ CD206- CD11c+ ROS	0,79	0,0188

Lactobacillus	MFI CD11b+ F4/80+ CD206- CD11c+ ROS	-0,84	0,0092
Lachnospiraceae GCA-900066575	MFI CD11b+ F4/80+ CD206- CD11c+ ROS	0,81	0,0147
Lactobacillaceae HT002	MFI CD11b+ F4/80+ CD206- CD11c+ ROS	-0,8	0,0178
Eubacterium nodatum group	MFI CD11b+ F4/80+ CD206- CD11c+ ROS	-0,71	0,0483
Lachnospiraceae ASF356	MFI CD11b+ F4/80+ CD206- CD11c+ ROS	-0,73	0,0412
Lachnospiraceae FCS020 group	MFI CD11b+ F4/80+ CD206- CD11c+ ROS	0,71	0,0493
Bilophila	MFI CD11b+ F4/80+ CD206- CD11c+ ROS	0,73	0,0385
Anaerovorax	MFI CD11b+ F4/80+ CD206- CD11c+ ROS	-0,74	0,0343
Peptococcus	MFI CD11b+ F4/80+ CD206- CD11c+ ROS	-0,92	0,0011
Christensenellaceae R-7 group	MFI CD11b+ F4/80+ CD206- CD11c+ ROS	-0,81	0,0141
Lachnospiraceae UCG-004	MFI CD11b+ F4/80+ CD206- CD11c+ ROS	-0,9	0,0022
Rikenellaceae RC9 gut group	% CD11b+ F4/80+ CD206+ CD11c+	0,74	0,0344
Negativibacillus	% CD11b+ F4/80+ CD206+ CD11c+	-0,9	0,0022
Harryflintia	% CD11b+ F4/80+ CD206+ CD11c+	-0,77	0,0264
Lachnospiraceae ASF356	% CD11b+ F4/80+ CD206+ CD11c+	-0,73	0,0392
Lachnospiraceae UCG-004	% CD11b+ F4/80+ CD206+ CD11c+	-0,74	0,0364
Lachnospiraceae FCS020 group	% CD11b+ F4/80+ CD206- CD11c-	0,9	0,0025
Bilophila	% CD11b+ F4/80+ CD206- CD11c-	0,77	0,0248
Salmonella	% CD11b+ F4/80+ CD206- CD11c-	0,93	8.00E-04
Negativibacillus	MFI CD11b+ F4/80+ ROS	-0,75	0,0308
Ligilactobacillus	MFI CD11b+ F4/80+ ROS	-0,8	0,0166
Anaeroplasma	MFI CD11b+ F4/80+ ROS	0,77	0,0239
Alistipes	MFI CD11b+ F4/80+ ROS	0,75	0,0327
Lactobacillus	MFI CD11b+ F4/80+ ROS	-0,83	0,0112
Lachnospiraceae GCA-900066575	MFI CD11b+ F4/80+ ROS	0,82	0,0136
Lactobacillaceae HT002	MFI CD11b+ F4/80+ ROS	-0,79	0,0208
Eubacterium nodatum group	MFI CD11b+ F4/80+ ROS	-0,74	0,0346
Lachnospiraceae FCS020 group	MFI CD11b+ F4/80+ ROS	0,8	0,0173
Bilophila	MFI CD11b+ F4/80+ ROS	0,78	0,0213
Oscillospiraceae NK4A214 group	MFI CD11b+ F4/80+ ROS	-0,72	0,0435
Anaerovorax	MFI CD11b+ F4/80+ ROS	-0,74	0,0375
Peptococcus	MFI CD11b+ F4/80+ ROS	-0,94	6.00E-04
Christensenellaceae R-7 group	MFI CD11b+ F4/80+ ROS	-0,82	0,0132
Lachnospiraceae UCG-004	MFI CD11b+ F4/80+ ROS	-0,86	0,0064
Lachnoanaerobaculum	MFI CD11b+ ROS	-0,71	0,0484
Negativibacillus	MFI CD11b+ ROS	-0,74	0,0344
Ligilactobacillus	MFI CD11b+ ROS	-0,8	0,0164
Anaeroplasma	MFI CD11b+ ROS	0,75	0,0308
Alistipes	MFI CD11b+ ROS	0,79	0,0209
Lactobacillus	MFI CD11b+ ROS	-0,81	0,0156
Lachnospiraceae GCA-900066575	MFI CD11b+ ROS	0,81	0,0149
Lactobacillaceae HT002	MFI CD11b+ ROS	-0,77	0,0255
Eubacterium nodatum group	MFI CD11b+ ROS	-0,81	0,0152
Lachnospiraceae FCS020 group	MFI CD11b+ ROS	0,8	0,0169
Bilophila	MFI CD11b+ ROS	0,78	0,0231
Oscillospiraceae NK4A214 group	MFI CD11b+ ROS	-0,74	0,0343

Anaerovorax	MFI CD11b+ ROS	-0,75	0,0315
Peptococcus	MFI CD11b+ ROS	-0,96	2.00E-04
Christensenellaceae R-7 group	MFI CD11b+ ROS	-0,79	0,0201
Lachnospiraceae UCG-004	MFI CD11b+ ROS	-0,86	0,0062
Lachnospiraceae NK4A136 group	% CD11b-	0,81	0,014
Herbinix	% CD11b-	0,76	0,0275
Anaeroplasma	% CD11b-	0,75	0,0323
Prevotellaceae NK3B31 group	% CD11b-	0,83	0,0111
Lachnospiraceae GCA-900066575	% CD11b-	0,8	0,0162
Angelakisella	% CD11b-	0,79	0,0197
Anaerosporobacter	% CD11b-	0,79	0,0206
Marvinbryantia	% CD11b-	0,85	0,0082
Rikenellaceae RC9 gut group	% CD11b- CD36+	0,89	0,0033
Bacteroides	% CD11b- CD36+	0,72	0,0431
Negativibacillus	% CD11b- CD36+	-0,83	0,0108
Parabacteroides	% CD11b- CD36+	0,86	0,0065
Harryflintia	% CD11b- CD36+	-0,72	0,0425
Lactobacillus	% CD11b- CD36+	-0,72	0,0444
Lachnospiraceae UCG-004	% CD11b- CD36+	-0,73	0,0387
Lachnoanaerobaculum	% Leukocytes CD36+	-0,71	0,0465
Rikenellaceae RC9 gut group	% Leukocytes CD36+	0,89	0,0029
Bacteroides	% Leukocytes CD36+	0,75	0,0309
Negativibacillus	% Leukocytes CD36+	-0,81	0,0157
Parabacteroides	% Leukocytes CD36+	0,87	0,0055
Harryflintia	% Leukocytes CD36+	-0,71	0,048
Lactobacillus	% Leukocytes CD36+	-0,76	0,0285
Lactobacillaceae HT002	% Leukocytes CD36+	-0,71	0,0469
Anaerovorax	% Leukocytes CD36+	-0,72	0,0439
Monoglobus	% Leukocytes CD36+	-0,72	0,0432
Lachnospiraceae UCG-004	% Leukocytes CD36+	-0,75	0,0307
Anaerovoracaceae Family XIII UCG-001	% Leukocytes CD36+	-0,73	0,0418
Negativibacillus	MFI Leukocytes ROS	-0,8	0,017
Ligilactobacillus	MFI Leukocytes ROS	-0,85	0,0071
Anaeroplasma	MFI Leukocytes ROS	0,77	0,0245
Alistipes	MFI Leukocytes ROS	0,82	0,0131
Lactobacillus	MFI Leukocytes ROS	-0,84	0,0094
Lachnospiraceae GCA-900066575	MFI Leukocytes ROS	0,82	0,0118
Lactobacillaceae HT002	MFI Leukocytes ROS	-0,79	0,0207
Eubacterium nodatum group	MFI Leukocytes ROS	-0,81	0,0158
Lachnospiraceae FCS020 group	MFI Leukocytes ROS	0,74	0,0366
Bilophila	MFI Leukocytes ROS	0,77	0,0245
Oscillospiraceae NK4A214 group	MFI Leukocytes ROS	-0,71	0,0484
Anaerovorax	MFI Leukocytes ROS	-0,74	0,0356
Peptococcus	MFI Leukocytes ROS	-0,91	0,0015
Christensenellaceae R-7 group	MFI Leukocytes ROS	-0,77	0,0241
Anaerostipes	MFI Leukocytes ROS	-0,73	0,0412

Lachnospiraceae UCG-004	MFI Leukocytes ROS	-0,84	0,0086

Supplementary Table S6: Correlation between body weight, blood metabolic parameters and immune cells present in VAT with bacterial genera of gut microbiota showing significant correlation. Correlation significant established using the Pearson test (Significant p-value<0.05 was consider).

Abbreviations: CD: cluster of differentiation, VAT: visceral adipose tissue, HDL-c: high-density lipoprotein cholesterol, LDL-c: low-density lipoprotein cholesterol, AST: aspartate aminotransferase, ALT: alanine aminotransferase, ROS: reactive oxygen species, MFI: median fluorescence intensity.
